# Intensive community and home-based treatments for eating disorders: a scoping review

**DOI:** 10.1186/s40337-025-01429-1

**Published:** 2025-11-10

**Authors:** Başak İnce, Amelia Austin, Matthew D. Phillips, Elizabeth Fordham, Erica Cini, Ulrike Schmidt

**Affiliations:** 1 Department of Psychological Medicine, Centre for Research in Eating and Weight Disorders (CREW), Institute of Psychiatry, Psychology, and Neuroscience, King’s College London, London, UK; 2 Mathison Centre for Mental Health Research and Education, Cumming School of Medicine, University of Calgary, Calgary, AB Canada; 3 Department of Community Health Sciences, Cumming School of Medicine, University of Calgary, Calgary, AB Canada; 4 University College London, London, UK; 5 Department of Child and Adolescent Psychiatry, Institute of Psychiatry, Psychology, and Neuroscience, King’s College London, London, UK; 6 East London NHS Foundation Trust, London, UK; 7 South London and Maudsley NHS Foundation Trust, London, UK

**Keywords:** Eating disorders, Home treatment, Intensive community treatment, Intensive care, Intensive outpatient, Home-based care

## Abstract

**Background:**

Intensive community treatment (ICT) and home-based treatment (HBT) have emerged as valuable alternatives to institution-based intensive treatments (inpatient or day patient) for severe mental illnesses. Although potential benefits of ICT and HBT for eating disorders (EDs) have been proposed, this area of research remains largely unexplored.

**Method:**

A scoping review was conducted to map the available literature. Four databases (PubMed, PsycInfo, MEDLINE, Web of Science), grey literature, and trial registries were searched. Sources were included if they presented treatments offering more than two planned therapeutic contacts per week for at least part of the program, excluding physical monitoring contacts, for patients diagnosed with any ED across all ages.

**Results:**

Forty-six sources met the inclusion criteria (ICT: n = 31; HBT: n = 15), with most studies from Europe (n = 23) and the USA (n = 18). Among these, 28 reported quantitative data, six reported qualitative data, and three employed a mixed-methods approach. The remainder were either protocol papers or service descriptions only. The majority focused on anorexia nervosa (AN) or mixed EDs, with varying study designs and predominantly low to moderate evidence quality. There were no randomized controlled trials. HBTs primarily targeted children and adolescents with AN, emphasizing family-based approaches, while ICTs exhibited greater variability in age groups and diagnoses, frequently combining cognitive behavioral and dialectical behavioral therapies, often alongside family-based components for children and adolescents. Despite high variability in design, quality, and measurements, studies frequently reported improvements in clinical outcomes. Programs were often described as feasible and acceptable, noting patient satisfaction, strong adherence, and cost-effectiveness due to reduced hospital admissions.

**Conclusions:**

Even though there was variability in implementation and methodologies, ICTs and HBTs appear to be promising alternatives to traditional institution-based intensive treatments. Future research requires higher-quality large-scale randomized trials with improved reporting of treatment characteristics and outcomes to enable robust investigations of effectiveness.

**Supplementary Information:**

The online version contains supplementary material available at 10.1186/s40337-025-01429-1.

## Abstract (plain language summary)

This scoping review aims to investigate intensive community-based (ICT) and home-based treatments (HBT) as alternatives to traditional hospital care for eating disorders (EDs). We searched four major databases to identify studies where patients with ED received more than two therapeutic contacts weekly for at least part of the program, excluding physical monitoring appointments. Forty-six sources were included, mostly from Europe and the USA. HBTs often focused on children and adolescents with anorexia nervosa, emphasizing family involvement. ICTs varied more by target age and diagnosis, frequently cognitive behavioral and dialectical behavioral therapies, often alongside family-based components for children and adolescents. Despite differences in study designs, these studies often reported improved patient outcomes, high satisfaction, and cost savings due to fewer hospital admissions. While the evidence quality is currently low to moderate with no randomized controlled trials, ICTs and HBTs seem to be promising interventions. Future research in this area needs to design higher-quality studies with clearer descriptions of treatment ingredients and more consistent reporting of how well treatments work.

**Supplementary Information:**

The online version contains supplementary material available at 10.1186/s40337-025-01429-1.

## Introduction

Eating disorders (EDs) are “disabling, deadly, and costly” psychiatric disorders characterized by persistent dysfunctional eating and/or weight-control behaviors [1] (p. 899). When not treated rapidly and effectively, EDs may follow a relapsing or chronic course with reduced likelihood of full recovery. The psychological, social, and economic burdens imposed by EDs are considerable, affecting not only the individual with the illness but also their caregivers and wider society [2].

People from all genders, ages, ethnicities, body types and weights, sexual orientations, and socioeconomic backgrounds are affected by EDs [3]. The prevalence of EDs has been continually rising across all age groups, with estimates in 2019 suggesting that globally about 55.5 million (95% UI, 38.7–75.2) have an ED [4–6]. Accompanying the rise in ED prevalence in the community, the need for hospital admissions is also increasing. For instance, ED-related hospital admissions in England doubled between 1998 and 2020 [7]. The COVID-19 pandemic has further escalated the number of people presenting with ED symptoms in the general population as well as heightening severity among those previously diagnosed with an ED [8, 9]. The impact of the COVID-19 pandemic led to an average increase in ED hospital admissions of 48% internationally [8]. Unsurprisingly, the rise in prevalence and severity of EDs have resulted in growing demand for access to specialist services and different forms of intensive treatments for EDs [4, 9, 10].

ED treatments often follow a stepped-care model, tailoring the intensity of treatment to match symptom severity and presentation. International evidence-based guidelines recommend specialized outpatient treatment as a first option for most, while more intensive treatment options (i.e., day patient/partial hospitalization, inpatient and residential care) are considered in cases where the person has not benefitted from outpatient treatment, or where there is high medical risk [11–15]. This stepped-care approach allows patients to start treatment at a lower intensity, progressing to more intensive care if deemed necessary to achieve recovery. This approach also facilitates transition in the other direction, from intensive institution/hospital-based care to outpatient care.

Higher level institution-based care including inpatient treatment (IPT), residential care (RC), partial hospitalization programs (PHP), and day patient treatments (DPT) are usually provided by multidisciplinary specialist ED services combining medical, psychological and nutritional support. These approaches vary in intensity, with IPT/RC being the most intensive and DPT/PHP often serving as a step-down from inpatient care, usually involving 6–10 h per day, 3–7 days per week. The structure, intensity, and labeling of these programs may vary across healthcare systems and countries. Family and caregiver involvement also often plays a vital role in these treatments across all age groups, enhancing treatment and providing a more comprehensive approach to care. These institution-based traditional intensive treatments have clear benefits, such as promoting recovery in a safe and supportive environment, offering regular meal support, restoring weight and managing medical risks and improving physical health [16–19]. IPT in particular may also provide families with much needed respite. Higher-level institution-based care separates patients from their everyday environments, a separation that is total in inpatient and residential programs and substantial in day patient or partial hospitalization settings. This can lead to notable challenges, such as risk of institutionalization, reduced patient autonomy, strict institutional schedules and routines, geographical inequality and disruptions to daily routines [19–21]. Furthermore, the cost of these treatments and risk of relapse following discharge are high [22–24].

To overcome the challenges of traditional institution-based intensive treatments, intensive community-based (ICT) and home-based treatments (HBT) have been proposed as feasible and potentially cost-effective alternatives. ICT provides treatment, care, and support in community settings (e.g., specialized intensive outpatient settings, schools, primary care), while HBT provides these services directly in the patient’s home, both of which enable patients and families to manage their illness either in their everyday environments or in a non-institutional setting.

The effectiveness of ICTs and HBTs has been investigated across a range of psychiatric disorders, including affective disorders, psychosis, personality disorders, and substance use disorders, and across different age groups. These approaches have been associated with improved clinical outcomes, increased treatment adherence, and reductions in hospital admissions, readmissions, length of stay, and overall treatment costs [25–28]. For instance a pragmatic randomized controlled trial involving 707 adults in need of immediate hospital admission for various mental health crises (e.g., schizoaffective disorder, bipolar disorder, personality disorder) found that home treatment resulted in 30.4% fewer hospital bed days over 24 months compared to standard inpatient care, with comparable clinical and social outcomes and similar levels of patient satisfaction [29]. Furthermore, two recent meta-analytic reviews support the effectiveness of ICTs and HBTs as alternatives to institution-based care: one showed no significant differences in psychosocial and psychopathology outcomes between HBT and inpatient care for children and adolescents with psychiatric disorders [30], and the other found similar reductions in PTSD symptoms between residential and intensive outpatient treatments in military populations [31].

With the growing body of research on alternatives to traditional institution-based intensive treatments for various psychiatric disorders showing encouraging findings, it is timely to assess their potential utility in EDs. Efforts to develop and investigate intensive community-based and home-based treatments, have been supported by recommendations from recent reports by BEAT, the UK's leading charity for ED patients and caregivers [32, 33].

However, despite the proposed advantages of ICT and HBT for the treatment of EDs, which could provide similar outcomes to IPT while reducing healthcare costs and being potentially more acceptable to patients and their families, a comprehensive review of research focusing on these treatments has not yet been conducted. Therefore, we aimed to map the available literature on intensive community and home treatments for EDs by addressing the following research questions:

1. What is the extent of the available literature on intensive community and home-based treatments for children, adolescents and adults with EDs?
2. How are intensive community and home-based treatments for EDs conceptualized and implemented across the age range?
3. What is the available evidence on the efficacy, acceptability, and cost-effectiveness of intensive community and home-based treatments for eating disorders (EDs)?

The third question was included to identify study designs, outcomes and gaps in the knowledge base, that together may inform the development of questions for a future systematic review [34].

## Method

A scoping review methodology was used in this study to explore the emerging knowledge on intensive community (ICT) and home-based treatments (HBT) for children, adolescents and adults with EDs. Details on the methodology of this review can be found in the published protocol [35]. This study was designed and conducted in compliance with the Preferred Reporting Items for Systematic Review and Meta-Analysis Protocol Extension for Scoping Reviews checklist (PRISMA-ScR) [36] and the Joanna Briggs Institute (JBI) Reviewer’s Manual [37]. The JBI Manual provides comprehensive methodological guidance for conducting scoping reviews, including when such reviews are appropriate, how to systematically extract, analyze, and present data, and how to interpret implications for practice and research. It is aligned with PRISMA-ScR and promotes methodological rigor and transparency, particularly in the assessment and coding of evidence.

### Eligibility criteria

The eligibility criteria for this review as outlined in the protocol were: (1) sources on individuals with EDs from all age and gender groups; (2) studies investigating ICT and HBT for EDs that offer more than two planned therapeutic contacts per week for at least a portion of their treatment protocol, excluding physical monitoring contacts; (3) sources using quantitative, qualitative, mixed methods, descriptive and case study methodologies and designs; (4) any type of evidence source, including peer-reviewed articles, book chapters, dissertations, theses, conference abstracts, non-peer-reviewed articles, and trial registries; and (5) publications in English. We made one slight change to the eligibility criteria since the publication of the protocol [35], which was to include studies focused on families/caregivers and healthcare providers (i.e., not just patients) impacted by or working in ICT or HBT. Additionally, the third research question was amended to identify and map the breadth of available evidence on the efficacy, acceptability and cost-effectiveness of ICTs and HBTs for EDs, to better align with scoping review methodology guidance [34, 38].

Exclusion criteria included: (1) sources on mixed psychiatric populations where no separate data for EDs are available; (2) describing non-intensive treatments (i.e., offering two or fewer therapeutic contacts per week); (3) describing institution/hospital-based treatments (i.e., inpatient and day patient); (4) not providing separate outcome for ICT and or HBT (i.e., presenting outcomes combined with other settings) and (5) review articles and meta-analyses.

### Search strategy

The search strategy adhered to the protocol [35]. In brief, a systematic literature search was conducted in four main databases (PubMed, PsycInfo, MEDLINE, Web of Science) and six grey literature databases (Scopus, Google Scholar, US ClinicalTrials.gov, WHO International clinical trials registry platform search portal, ISRCTN Registry, and the European Union Clinical Trials Register). An additional hand search was conducted on relevant journals and reference lists of papers in this review. The search terms included variations of “feeding and eating disorders”, “community treatment”, “intensive outpatient”, and “home treatment”. The exact search terms are presented in the supplemental material 1. The cut-off date for the searches in the databases was 13th of January 2025.

### Study selection

Identified studies were imported into Endnote citation management software [39] and duplicates were removed. The references were then transferred to Rayyan, a systematic review management software [40], where title, abstract and full-text screening took place. For conference abstracts where no full text was readily available (n = 4), we contacted the corresponding authors to inquire about the full texts. Three responded, and we were able to obtain the full texts from two of these. All titles, abstracts and full texts of identified sources were screened by at least two researchers. Discrepancies between raters were resolved by discussion, and when necessary, a third rater conducted an independent screening.

### Data charting, extraction, and analysis

The data charting form was developed by the lead author in consultation with the co-authors (Please see Supplemental material 2). Subsequently, data were extracted and charted into a structured table within an Excel spreadsheet, aligning with the objectives of this review. A minimum of two researchers independently completed data extraction and charting for each source included. Extracted data included study details (authors, year, country, design, setting), participant characteristics (sample size, diagnosis, age, sex, gender, gender identity, race/ethnicity, socioeconomic status), treatment specifics (programs, population, admission criteria, treatment model, meal support, length, intensity, delivery mode, professionals involved, carer/family involvement), ED-related outcomes (e.g., body mass index, changes in symptoms), feasibility, acceptability, cost-effectiveness, and qualitative outcomes.

A narrative synthesis approach was adopted for analysis due to heterogeneity in the methodologies employed across sources. For each included source, we characterized the types of evidence using the JBI Levels of Evidence, which categorize study designs and assess the strength of evidence across domains of (i) effectiveness, (ii) meaningfulness, and (iii) economic evaluations [41].

## Results

The results are presented in accordance with the research questions, with a specific focus on mapping the body of literature across different age groups (children and adolescents, adults, and mixed-age populations) to explore similarities and differences in the available research.

### Extent of the literature

**Fig. 1:**
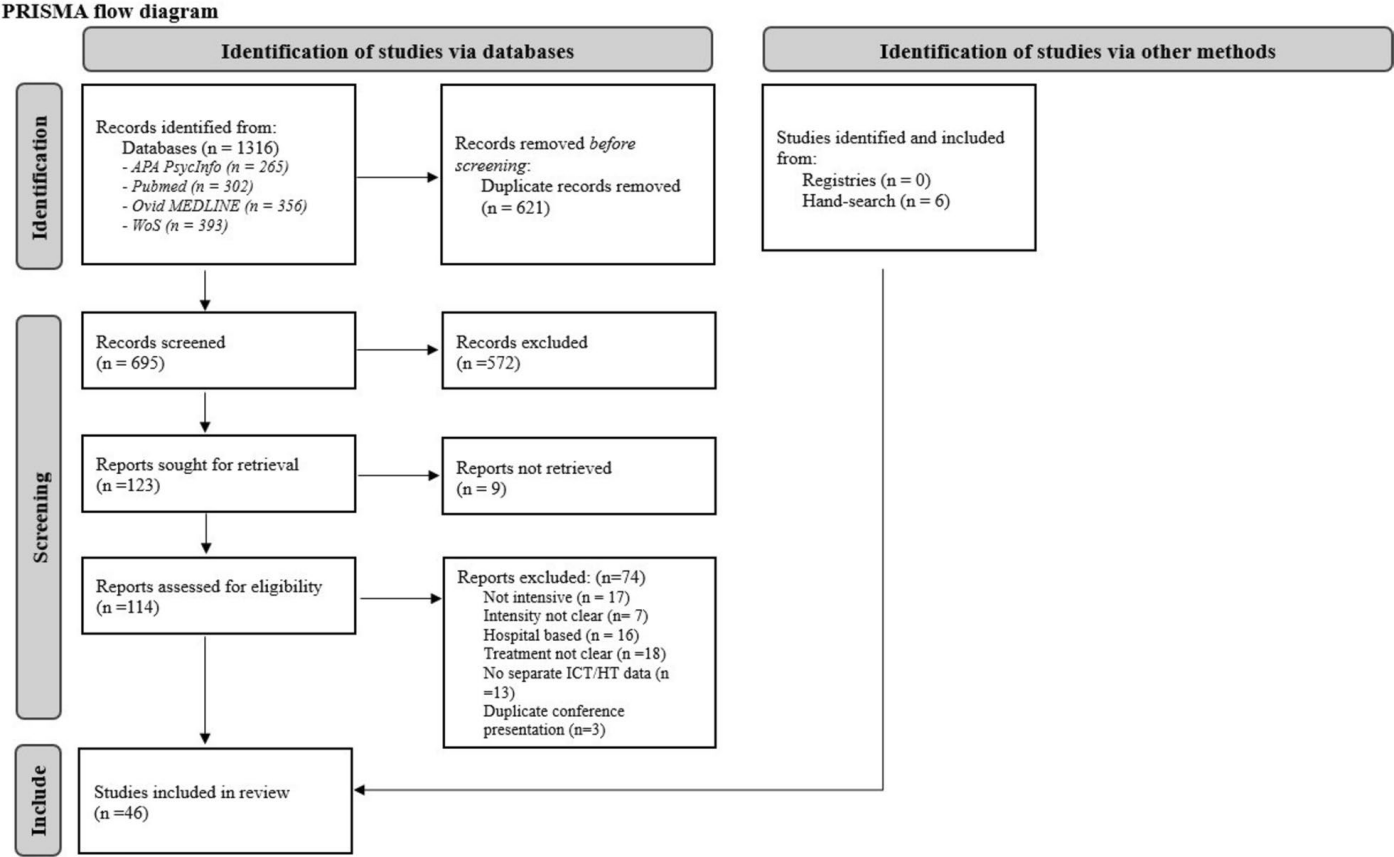
PRISMA flow diagram

After removal of duplicates, a total of 695 unique sources were identified for screening from database searches, grey literature, and hand searches. Nine papers were directly excluded during title and abstract screening and were not evaluated for inclusion criteria, as full texts in English were unavailable. Abstracts of these sources are presented in Supplemental material 3. Full text of 114 sources were screened, and among those 46 sources were included in this review (see Fig. 1 for PRISMA flowchart), with 31 of these focusing on intensive community treatments (ICT) and 15 on home-based treatments (HBT). When presenting the treatments, we kept the terminology used by the original sources whenever possible. For programs offering a mixture of ICT and HBT, we categorized them according to the primary treatment setting described.

Most of the 46 included sources, originated from Europe (n = 23), followed by the USA (n = 18), Canada (n = 3), Colombia (n = 1), and Israel (n = 1). While the majority (12 out of 15) of HBT programs took place in Europe, the distribution of ICT was more geographically diverse. The majority of the included sources were published in peer-reviewed journals (n = 35), while seven were unpublished theses/dissertations, three were conference abstracts, and one was an unpublished paper.

Participants’ baseline sociodemographic characteristics are presented in Table 1. In brief, the studies focusing on children and adolescents (n = 22) and adults (n = 17) were similar in numbers. Fourteen sources included only female participants, while only three reported on inclusion of non-binary and/or transgender individuals. Participants and/or target population in the sources included were primarily diagnosed with anorexia nervosa (AN; n = 16) or a mixture of different EDs (n = 18). Reporting on socioeconomic status (n = 6) and ethnicity/race (n = 15) was limited.

**Table 1:** Details of included sources and sociodemographic characteristics of participants

Author(s)	Country	Sample size	Mean/median age at baseline	Sex, gender, and gender identity*	Race and ethnicity	Socioeconomic status
**Home-based treatments**						
Besse-Flütsch et al. [42]	Switzerland	90 adolescent patients + parents/caregivers (planned)	12–18 (planned)	NA	NA	NA
Bezance and Holliday [43]	UK	9 mothers of adolescent patients	Patients: 13–16 Mothers: 40–63	NR	NR	NR
Clark-Stone et al. [44]	UK	33 adolescent patients + 6 parents	M = 14 years 7 months Range: 11 years and 10 months to 17 years and 10 months	Female: 100%	NR	NR
Dahmen et al. [46]	Germany	240 patients (planned)	12–18 (planned)	NA	NA	NA
Daniel et al. [64]	France	(i) Tube feeding (TF) + CBT: 61 (ii) Tube feeding (TF) alone: 57	(i) TF + CBT: M(SD) = 26.7 (6.4) (ii) TF alone = 28.6 (6.6)	Female: 100%	NR	NR
Flütsch et al. [45]	Switzerland	45	M(SD) = 15.6 (1.8) Range: 10.7–19.6	Female: 95.5% (n = 3) Male: 4.5% (n = 2)	NR	NR
Goldschmidt et al. [48]	USA	NA	12–18 (planned)	NA	NA	NA
Goldschmidt et al. [47]	USA	NA	NA	NA	NA	NA
Heider et al. [49]	Germany	21	M(SD) = 15.10 (1.16) Range: 12–18	Female: 100%	NR	NR
Herpertz-Dahlmann et al. [50]	Germany	22	M(SD) = 15.06 (1.15) Range: 13.17- 17.03	Female: 100%	NR	NR
Latzer et al. [51]	Israel	3	Ages: 13; 15; 16.5	Female: 100%	NR	NR
Mayr et al. [52]	Switzerland	61	M(SD) = 15.6 years (1.8) Range: 10.7–19.6 years	Female: 95.1% 58 (n = 58) Male: 4.9% (n = 3)	NR	NR
Morón-Nozaleda et al. [53]	Spain	59	M (SD) = 14.69 (1.67)	Female: 100%	NR	NR
Pauli et al. [54]	Switzerland	(i) HBT + FBT: 45 (ii) FBT only: 22	(i) HBT + FBT: M(SD) = 15.6 (1.8) (ii) FBT only: M(SD) = 15.6 (1.8)	Female: 95.5% (n = 63) Male: 4.5% (n = 3)	NR	NR
Tsiaka and Bletsos [81]	Greece	10	14–35 years	NR	NR	NR
**Community-based treatments**						
Blalock et al. [82]	USA	57 patients	M(SD) = 29.91 (11.91)	Female: 96% (n = 53)	White-Caucasian: n = 44 (80%) Other: n = 13 (20%)	NR
Chiumiento [55]	USA	205 patients + 289 parents	M(SD) = 15 (1.6) Range: 11–19	Female: 92% (n = 266) Male: 8% (n = 23)	NR	NR
Crenshaw [65]	USA	10	Median = 39 Range: 27–59	Female: 100%	White-Caucasian: n = 8 (80%) Jewish-Caucasian: n = 1 (10%) Black: n = 1 (10%)	1 identified as lower middle class, 7 as middle class and 2 as upper middle class
Deumens et al. [66]	Netherlands	182	M(SD) = 35.1 (8.5) Range: 18–57	Female: 100%	NR	NR
Doyle et al. [56]	USA	44	Range: 10–18	Female: 100%	NR	NR
Federici et al. [67]	USA	NA	NA	NA	NA	NA
Hannon et al. [68]	UK	5	Range: 23–30	Female: 100%	100% White-Caucasian	Socio-economic status is reflected by the Cairstairs Index, used to describe levels of deprivation in the Scottish population (Carstairs & Morris, 1991). This generates five categories ranging from 1 (most deprived) to 5 (least deprived). Two participants were in category 2, two in category 3 and one in category 5
Johnston et al. [57]	USA	51	M(SD) = 14.8 (1.5) Range: 12–17.5	Female: 100%	NR	NR
Kim [58]	USA	36	M(SD) = 14.57 (1.93) Range: 12–19	Female: 94.4% (n = 34) Male: 5.6% (n = 2)	91.7% White (n = 33) 5.5 Asian (n = 2) 2.8% Other (n = 1)	NR
Komarova [59]	UK	5 clinicians	NA	NA	NA	NA
Kuang et al. [60]	UK	32	M (SD) = 13.6 (0.3) Range: 11–17	Female: 90.6% (n = 29) Male: 9.4% (n = 3)	50% White (n = 16) 34.4% Asian/Asian British (n = 11) 9.4% Mixed (n = 3) 6.3% Other (n = 2)	*Average household income by local area* £26 k-31 k: 18.8% (n = 6) £31 k-36 k: 37.5% (n = 12) £36 k-43 k: 31.3% (n = 10) £43 k-67 k: 12.5% (n = 4)
Lammers et al. [71]	Netherlands	431	M(SD) = 36.38 (9.35) Range: 18–60	Female: 92.6% (n = 399) Male: 7.4% (n = 32)	NR	NR
Lammers et al. [70]	Netherlands	(i) CBT + : 33 (ii) DBT-BED: 41	M(SD) = 37.3 (11.8) Range: 18–67	Female: 89.2% (n = 66) Male: 10.8% (n = 8)	NR	NR
Lammers et al. [69]	Netherlands	(i) CBT + : 133 (ii) DBT-BED: 42	(i) CBT + : M(SD) = 33.46 (10.75) (ii) DBT-BED: M(SD) = 39.40 (10.22)	(i) CBT + Female: 88.7% (ii) DBT-BED Female: 90.5%	NR	NR
Levinson et al. [72]	USA	(i) In-person: 60 (ii) Telehealth: 33	(i) In-person: M(SD) = 25.07 (7.88) (ii) Telehealth: M(SD) = 24.52 (9.27)	(i) In-person: Female: 91.67% (n = 55) (ii) Telehealth: Female: 90.91% (n = 30)	(i) In-person 98.33% White 1.67% Black (ii) Telehealth 90.91% White 3.03% Black 3.03% Asian 3.03% Multiracial/biracial	NR
Lowe et al. [83]	USA	NA	Range: 14–55	Female: 100%	NR	NR
Lui [73]	USA	1	35	Female: 100%	Latina American	NR
MacDonald et al. [75]	Canada	(i) IOP Group: 103 (ii) Individual CBT: 118	(i) IOP group: M(SD) = 28.4 (9.2) (ii) Individual CBT: M(SD) = 28.6 (8.6)	(i) IOP Group Female: 97.1% Male: 1.9% Transgender Individuals: 1.0% (ii) Individual CBT Group Female: 94.1% Male: 5.9%	(i) IOP Group 85.4% White Caucasian 2.9% African-Canadian/Black 4.9% Asian 1.0% Latinx 2.9% Mixed 2.9% Other (ii) Individual CBT Group 76.4% White 2.7% African-Canadian/Black 5.5% Asian 0.0% Latino/a6.4% Mixed 9.1% Other	NR
MacDonald et al. [74]	Canada	(i) IPT + IOP: 9 (ii) IPT + individual therapy: 6 (iii) IOP alone: 3 (iii) Individual therapy alone: 12	M (SD) = 26.0 (6.9) Range: 17- 48	Cisgender Female: 61.9% (n = 26) Cisgender Male: 21.4% (n = 9) Transgender/non-binary/other gender identity: 16.7% (n = 7)	White: 65.9% (n = 27) Asian: 7.3% (n = 3) Indigenous: 4.9% (n = 2) Black, middle eastern or other: 17.1 (n = 7) Biracial/multiracial: 4.9% (n = 2)	Financial support (n = 39) Self-supporting 23.1% 9 Partially self-supporting 33.3% 13 Completely dependent (on partner, family, or government) 43.6% 17 Educational attainment (n = 41) Below high school 22.0% 9 High school 41.5% 17 College diploma 14.6% 6 Undergraduate degree 14.6% 6 Professional or graduate degree 7.3% 3
Monk [61]	USA	137	M(SD) = 14.8 (1.83) Range: 10–19	Female: 89.1% (n = 122) Male: 10.9% (n = 15)	88.3% White-Caucasian 1.5% Caucasian + Latino 0.7% Caucasian + African American 2.2% Latino 2.2% Asian 0.7% African American 3.6% Other	Median annual household income ranged from $27,652 to $180,607, with a mean of $89,450.89 (SD = 26,024.47)
Munro et al. [77]	UK	33	NR	NR	NR	NR
Munro et al. [76]	UK	26	M (SD) = 26.8 (9.7) Range: 18–52	Female: 96% (n = 25)	NR	NR
Novack et al. [62]	Canada	NA	12–18 (planned)	NA	NA	NA
Rienecke et al. [84]	USA	(i) Adults: n = 305 (ii) Children and adolescents: n = 33	(i) Adults: 29.63 (10.95) Range: 18–63 (ii) Children and adolescents: 14.73 (1.35) Range: 11–17	(i) Adults: Cisgender Men (n, %) = 12 (3.9%) Cisgender Women (n, %) = 279 (91.5%) Nonbinary (n, %) = 6 (2.0%) Transgender Men (n, %) = 1 (0.3%) Declined to answer (n, %) = 7 (2.3%) (ii) Children and adolescents: Cisgender Men (n, %) = 3 (9.1%) Cisgender Women (n, %) = 28 (84.8%) Nonbinary (n, %) = 2 (6.1%)	(i) Adults: Asian (n, %) = 10 (3.3%) Black/African American (n, %) = 8 (2.6%) Hispanic/Latino (n, %) = 17 (5.6%) White (n, %) = 253 (83.0%) 1 (3.0%) Unknown (n, %) = 12 (3.9%) (ii) Children and adolescents Asian (n, %) = 1 (3.0%) Hispanic/Latino (n, %) = 3 (9.1%) White (n, %) = 26 (78.8%) Biracial (n, %) = 1 (3.0%) Unknown (n, %) = 2 (6.1%)	NR
Rodríguez Guarin et al. [85]	Colombia	14 patients + 10 family members + 8 clinicians	Range: 13–20	NR	NR	NR
Saeidi et al. [78]	UK	6 patients + 8 clinicians	NR	NR	NR	NR
Van Huysse et al. [63]	USA	(i) In-person PHP: 49 (ii) Virtual IOP: 53	In-person PHP: M (SD) = 15.00 (2.67) Virtual IOP: M (SD) = 15.28 (2.26)	In-person PHP: Female: 84% Male: 16% Virtual IOP: Female: 93% Male: 8%	*In-person PHP* *Race:* 94% white, 2% Black or African American, 2% other *Ethnicity:* 10% Hispanic or Latinx, 90% Not Hispanic or Latinx *Virtual IOP Race:* 94% White, 2% Black or African American, 4% Other *Ethnicity:* 4% Hispanic or Latinx, 96% Not Hispanic or Latinx	NR
Vroling et al. [79]	Netherlands	376	M (SD) = 36.5 (10.21) Range: 18–61	Female: 92.3% (n = 347) Male: 7.7% (n = 29)	NR	NR
Walker et al. [86]	USA	210	M (SD) = 25.10 (10.43) Range: 13–62	Female: 89.4%	94.2% White 1.3% Hispanic/Latino 3.9% Asian 0.6% Mixed Race	NR
Wilkes [80]	USA	34	M (SD) = 35.65 (13.73)	Female: 100%	82% White (n = 28) 11% Mixed-race (n = 4) 6% Black or African American (n = 2)	Family income: 23.5% < $50,000, 35.3% between $50,000–80,000, 14% between $80,000–100,000, 11.8% between $100,000–150,000 and 14.7% > 150,000
Wolfe et al. [87]	USA	38	NR	NR	NR	NR

The wordings used in the table are directly taken from the included sources

BED: binge eating disorder; CBT: cognitive behavioral therapy; DBT: dialectical behavior therapy; HBT: home-based treatment; IOP: intensive outpatient program; IPT: inpatient treatment; M (SD): mean (standard deviation); NA: not available; NR: not reported; PHP: partial hospitalization program; TF: tube feeding; UK: United Kingdom; USA: United States of America

The methodologies and designs were diverse (Table 2). Out of the 46 sources included, nine did not present any outcome data due to being protocol papers (n = 4), service descriptions (n = 3), or descriptive reports (n = 2). Among the remaining 37 studies, the majority employed quantitative methods (n = 28), with study designs primarily consisting of pilot studies, naturalistic studies and case reports/series. Most (n = 28) included patient data only. A few incorporated perspectives from both patients and family/caregivers (n = 5) or patients and healthcare professionals (n = 1), while one study included all three groups. Additionally, one study focused solely on healthcare professionals, and another on mothers’ experiences. Sample sizes varied widely, ranging from 3 to 118 in HBT studies and 1 to 431 in ICT studies.

**Table 2:** Overview of study designs, settings, and levels of evidence

Author(s)	Setting	Study design	Data type	Levels of evidence for effectiveness	Levels of evidence for meaningfulness	Levels of evidence for economic evaluation
Besse-Flütsch et al. [42]	HBT	RCT (protocol)	Quantitative	NA	NA	NA
Bezance and Holliday [43]	HBT	Naturalistic study (retrospective)	Qualitative	NA	3	NA
Blalock et al. [82]	ICT	Naturalistic pilot feasibility study	Quantitative	3e	NA	NA
Chiumiento 2016 [55]	ICT	Secondary analyses of archival data	Quantitative	4b	NA	NA
Clark-Stone et al. [44]	HBT	Service evaluation	Mixed	3e	3	NA
Crenshaw [65]	ICT	Naturalistic Study (retrospective)	Qualitative	NA	3	NA
Dahmen et al. [46]	HBT	RCT (protocol)	Quantitative	NA	NA	NA
Daniel et al. [64]	HBT	Open prospective study	Quantitative	3c	NA	NA
Deumens et al. [66]	ICT	Naturalistic study	Quantitative	3e	NA	NA
Doyle et al. [56]	ICT	Secondary analyses of archival data	Quantitative	3c	NA	NA
Federici et al. [67]	ICT	Service description	NA	NA	NA	NA
Flütsch et al. [45]	HBT	Case series/pilot study	Quantitative	3d	NA	NA
Goldschmidt et al. [48]	HBT	Pilot study (protocol)	Mixed	NA	NA	NA
Goldschmidt et al. [47]	HBT	Service development/description	Descriptive	4d	NA	NA
Hannon et al. [68]	ICT	Naturalistic Study	Qualitative	NA	3	NA
Heider et al. [49]	HBT	Single-centre, non-randomised, open-label pilot study	Quantitative	3e	NA	NA
Herpertz-Dahlmann et al. [50]	HBT	Single-centre, non-randomised, open-label pilot study	Quantitative	3e	NA	6
Johnston et al. [57]	ICT	Naturalistic pilot study	Quantitative	3e	NA	NA
Kim [58]	ICT	Secondary analyses of archival data	Quantitative	3e	NA	NA
Komarova [59]	ICT	Service evaluation	Qualitative	NA	3	NA
Kuang et al. [60]	ICT	Service evaluation	Quantitative	3e	NA	NA
Lammers et al. [71]	ICT	Naturalistic study	Quantitative	3e	NA	NA
Lammers et al. [70]	ICT	Open, quasi-randomized, controlled trial	Quantitative	1d	NA	NA
Lammers et al. [69]	ICT	Open, quasi-randomized, controlled trial	Quantitative	2c	NA	NA
Latzer et al. [51]	HBT	Case series	Descriptive	4d	NA	NA
Levinson et al. [72]	ICT	Nonrandomized control trial	Quantitative	2d	NA	NA
Lowe et al. [83]	ICT	Service description	NA	NA	NA	NA
Lui [73]	ICT	Case study	Quantitative	4d	NA	NA
MacDonald et al. [75]	ICT	Sequential cohort study	Quantitative	2d	NA	NA
MacDonald et al. [74]	ICT	Retrospective	Quantitative	3c	NA	NA
Mayr et al. [52]	HBT	Non‐randomized pilot study	Quantitative	2c	NA	6
Monk [61]	ICT	Secondary analyses of archival data	Quantitative	4b	NA	NA
Morón-Nozaleda et al. [53]	HBT	Retrospective feasibility study	Quantitative	3e	3	6
Munro et al. [77]	ICT	Service description	Mixed	4b	3	6
Munro et al. [76]	ICT	Naturalistic study	Quantitative	3e	NA	NA
Novack et al. [62]	ICT	Naturalistic study (protocol)	Quantitative	NA	NA	NA
Pauli et al. [54]	HBT	Waiting list control design pilot study	Quantitative	2c	NA	NA
Rienecke et al. [84]	ICT	Naturalistic study	Quantitative	4b	NA	NA
Rodríguez Guarin et al. [85]	ICT	Cross-sectional descriptive observational study	Qualitative	NA	3	NA
Saeidi et al. [78]	ICT	Service evaluation	Mixed	4c	3	6
Tsiaka and Bletsos [81]	HBT	Service description	NA	NA	NA	NA
Van Huysse et al. [63]	ICT	Naturalistic cohort study	Quantitative	2d	NA	6
Vroling et al. [79]	ICT	Naturalistic cohort study (secondary analysis)	Quantitative	4b	NA	NA
Walker et al. [86]	ICT	Naturalistic study	Quantitative	3e	NA	NA
Wilkes [80]	ICT	Naturalistic feasibility study	Quantitative	3e	NA	NA
Wolfe et al. [87]	ICT	Secondary analyses of data	Qualitative	NA	3	NA

ICT: intensive community treatment; HBT: home based treatment; NA: not applicable: RCT: randomized controlled trial

Evidence levels are based on the JBI Levels of Evidence. For effectiveness, Level 1 includes experimental designs (e.g., 1a: high-quality RCTs or systematic reviews), Level 2 quasi-experimental designs, Level 3 observational-analytic designs, Level 4 observational-descriptive studies, and Level 5 expert opinion and bench research. Sublevels (e.g., 1a, 2c) indicate further differentiation within a category based on study design or quality. For meaningfulness, levels range from 1 (highest) to 5 (lowest), and for economic evaluations, from 1 (highest) to 7 (lowest)

### Conceptualization and implementation of the treatment programs

Details of the programs are presented in Table 3.

**Table 3:** Overview of treatment programs

Treatment programs and author(s)	Treated population	Admission criteria	Underlying treatment model(s)	Meal support/supervised meal	Length and intensity	Delivery mode	Professional(s) delivering the intervention	Career/family involvement
*Programs for children and adolescents*								
Home-based treatments								
Department of Child and Adolescent Psychiatry, University of Zürich [Zürich, Switzerland] Flütsch et al. [45], Pauli et al. [54], Besse-Flütsch et al. [42], Mayr et al. [52]	Adolescents AN and AN atypical	Flütsch et al. [45], Pauli et al. [54], Mayr et al. 2024: Symptoms consistent with ICD-10 AN or atypical AN Besse-Flütsch et al. [42]: (1) Living with at least one adult caregiver (2) Willingness and ability to engage in family therapy (3) Being medically stable for outpatient treatment, as determined by a physician (4) Lack of comorbidities that contraindicate psychotherapy (e.g., psychosis) (5) An IQ greater than 75 (as determined by testing/clinical impression by a professional) (6) Adequate German language skill (7) Residency within the Canton of Zurich	(i) HBT + FBT (ii) FBT only (iii) FBT-MBSR (Besse-Flütsch et al. [42] only)	Yes (e.g., food preparation, serving, avoidance of discussion about portions)	(i) HBT + FBT: 1–4 × 60 min sessions per week + additional regular outpatient treatment (ii) FBT only: 1 × 60 min per week (iii) MBSR: 2 × 30-min per week (Besse-Flütsch et al. [42] only) *A period of 12 weeks*	In person & family	Flütsch et al. [45]; Pauli et al. [54], Mayr et al. 2024: Nurses (specialized in adolescent clinical healthcare) supervised by FBT ED clinician Besse-Flütsch et al. [42]: Graduate nurses or social workers with graduate professional degrees and experience in treatment of adolescent EDs	Yes (vital) To provide advice on overcoming key issues between the patient and parents, especially during mealtimes (e.g., food preparation, serving, and avoiding discussions about portions) To support refeeding efforts and foster family resilience in everyday social activities like hobbies and friendships
North Buckinghamshire Child and Adolescent Mental Health Service [Aylesbury, UK] Bezance and Holliday [43]	Adolescents AN & AN atypical	(1) Adolescent must have been referred from CAMHS to HBT (2) Adolescent had a primary diagnosis of AN or EDNOS-AN	FBT	Yes (supervision of meals at home)	3–5 contacts per week of around 60 min *Approximately 8 weeks (varied from 2 to 12 weeks)*	In person & family + additional individual support	A multidisciplinary team consisting of psychiatric nurses, occupational therapists, and clinical psychologists with psychiatry input	Yes (vital) To equip parents with the skills to support mealtimes at home, including supervision and practical support To provide emotional support to parents, helping them restore their resources and regain control
Child and Adolescent Home Treatment Team [Gloucestershire, UK] Clark-Stone et al. [44]	Children and adolescents Any EDs	(1) Young people under the age of 18 with severe EDs who are at risk of being admitted to a specialist inpatient unit, or who are returning home from hospital *OR* Parents are struggling to implement key principles of FBT (2) Young person does not attend school (3) Start of FBT at least a few weeks before referral to HBT	FBT + CRT + motivational exercises	Yes (modelling & supporting meals at home)	Starting with 10 home visits spread over 5 days a week and gradually reducing *A period of 6 weeks*	In person & family	Nurse/clinician	Yes (vital) To model how to support the young person during meal and snack times, showing parents how to manage their child’s eating and weight gain To guide parents on being empathetic yet firm, challenging disordered behaviors, and maintaining motivation during meals
[Pittsburgh -Pennsylvania; Providence & Pawtucket, Rhode Island, USA] Goldschmidt et al. [48], Goldschmidt et al. [47]	Adolescents AN & AN atypical	Study inclusion criteria: (1) Eligible for home-based treatment in a community-based clinic (2) Living with at least one adult caregiver who is willing and able to engage in family treatment (3) Medically stable for outpatient treatment per physician assessment (4) Free of comorbid conditions contraindicating psychotherapy or affecting weight or appetite (5) Not pregnant or lactating	(i) Manualized FBT + distress tolerance and emotion regulation skills (ii) TAU (supportive family therapy approach including psychoeducation and elements of CBT + DBT)	Yes (clinician assistance preparing and supervising meals with the adolescent meal planning, and grocery shopping)	(i) Manualized FBT: 3–6 h of weekly care, over 10–16 weeks (ii) TAU: 1–2 h sessions occurring at least 2 × per week *Across Rhode Island and Pittsburgh sites: 3–6 h of therapy per week. In Rhode Island sites, treatment is generally 6–12 weeks, and in Pittsburgh families are eligible for up to 32 weeks*	In person & family	Clinicians—not specified	Yes (vital) To model meal planning, preparation, and supervision, empowering caregivers in their refeeding efforts
Department of Child and Adolescent Psychiatry, Psychosomatics and Psychotherapy of the RWTH Aachen [Aachen, Germany] Herpertz-Dahlmann et al. [50], Heider et al. [49], Dahmen et al. [46]	Adolescents AN & AN atypical	(1) A diagnosis of AN or atypical AN according to DSM-5 (2) Age ≥ 12 years and ≤ 18 years (3) First or second admission for AN (4) Living with at least one carer within a commute of 60 min (5) Informed consent/assent of carers and patients	CBT + FBT	Initial IPT: Yes Herpertz-Dahlmann et al. 2020 & Heider et al. 2021 HBT: NR Dahmen et al. 2024: HBT: nutritional therapy	During the first two months, 3–4 × visits per week, over the third and 4th month 1–2 × visit per week, including a weekly AN-focused group psychotherapy session and family session *HBT: A period of 4 months with an average duration of M (SD) = 15.5 (1.2) weeks* *Initial IP admission—minimum of 4 weeks	In person & family + group + individual	A multidisciplinary team of an experienced nurse, a nutritional therapist, an occupational therapist and their individual therapist (psychotherapist or child and adolescent psychiatrist)	Yes (vital) To support parental management of food intake and eating disorder symptoms, facilitating weight gain
Mayanei HaYeshua Medical Center [Bnei Brak, Israel] Latzer et al. [51]	Adolescents AN	NR	Psychodynamic Psychotherapy + CBT	Yes	A weekly online meeting of the entire team staff with the patient and parents; daily monitoring to track pharmacotherapy, physiological and emotional condition, everyday functioning, and emerging difficulties; twice-weekly nutritional counselling; twice-weekly individual psychotherapy and once-weekly family therapy or parental guidance; a once-weekly psychiatric evaluation; group treatment; daily schooling by the educational staff for adolescent patients and continued rehabilitative care for young -adult patients by the occupational therapist and the social worker *Length not reported	Virtual & individual + family	A child and adolescent psychiatrist, an adult psychiatrist, a pediatrician, psychotherapists, nursing staff, clinical nutritionists, school staff, occupational therapists, spiritual therapists and support staff for the supervision of eating	Yes (vital)
Hospital Infantil Universitario Niño Jesús [Madrid, Spain] Morón-Nozaleda et al. [53]	Adolescents Any EDs	*Inclusion criteria:* (1) Diagnosis of an ED according to DSM-5 in patients who met severity criteria indicating hospitalization (2) Patient commitment to follow the program instructions (3) 24/7 availability for home care by at least one caregiver (4) A commute of ≤ 30 min from home to hospital (5) Acceptance to participate by the patient and legal guardians	CBT + FBT	Yes (supervised meals directly in the home)	Daily home visits from Monday to Friday approximately 30–60 min + 2 programmed calls per day *Mean stay of 39.14 days (SD = 14.47)*	In person & family + group	A multidisciplinary team of experts in ED, including a child and adolescent psychiatrist, clinical psychologist, nursing team, and pediatricians	Yes (vital) To provide psychoeducation, reinforcing the parental role and offering written instructions on meal planning, rest, and supervision To train parents in communication techniques and problem-solving strategies to help reduce anxiety
Community-based treatments								
The Walden Adolescent Intensive Outpatient Program [MA, USA] Chiumiento [55], Doyle [56], Johston et al. [57], Kim [58], Monk [61]	Adolescents Any EDs	(1) Aged between 12 and 17 years (2) Be medically stable (3) Have at least one parent able to commit to program attendance	FBT (Maudsley Approach) + CBT + DBT	Yes (supervised Multifamily dinner)	3 × 3-h group therapy sessions per week *A period of 8 weeks, or 24-day period*	In person & group + family	Clinicians—not specified	Yes (vital) To focus family sessions on enabling parents to take control of their child’s food intake, providing nutritional guidance and coaching during meal preparation To teach parents core DBT concepts and skills, focusing on family dynamics and communication
Eating Disorder Intensive Pathway—East London Foundation Trust [London, UK] Komarova [59]; Kuang et al. [60]	Children and adolescents Any EDs	Admission criteria for patients: (1) Aged under 18 years (2) Diagnosed with an ED (3) Live and have a GP within the three boroughs that EDIP operates across (4) Lack of progress in the current ED treatment and/or high risk of requiring admission to specialist eating disorder units (5) 'In-reach' to facilitate earlier discharge Study inclusion criteria for clinicians: (1) Healthcare professionals who work directly with patients in the EDIP	FT-ED (Maudsley Approach) + distress tolerance + CBT Mainly IOP but may also include HT and outreach	Yes	An average of 5 h per week; a maximum of 24 contacts per week *A period of 6 to 8 weeks*	In-person ± virtual ± hybrid	Pediatric and mental health nurses, pediatricians, psychiatrists, psychologists, support workers and dietitians	Yes (vital)
Intensive Ambulatory Care Program [Quebec, Canada] Novack et al. [62]	Adolescents Any EDs	*Study eligibility:* (1) Aged between 12 and 18 years (2) Have a diagnosed ED according to the DSM-5 (3) Have received medical treatment in the hospital or ambulatory setting at the specialized ED clinic (4) Must be available to participate in all aspects of the proposed intervention	FBT + CBT + DBT + motivational and psychoeducational approaches	Yes	3 to 4-h sessions per week *A period of 6 to 8 weeks*	Hybrid (web-based and in-person) & individual + group	Doctors, psychologists, social workers, etc	Yes (vital) To invite parents to participate in interventions addressing physical and psychological aspects of the disorder, including meal accompaniment, stress management, and family life To offer meetings both in-person and online for parental support
Michigan Medicine Comprehensive Eating Disorders Program [Michigan, USA] Van Huysse et al. [63]	Adolescents Any EDs	(1) Moderate to severe ED symptoms + functional impairment (2) Caregiver available to participate in treatment (3) Have had an unsuccessful trial of outpatient treatment and/or an acuity suggesting that outpatient is unsafe or unsuccessful, and/or unable to access appropriate outpatient treatment	FBT (primary) + CBT + DBT	Yes (only in PHP)	(i) PHP: 6 h per day for 5 days a week *Calendar days from treatment initiation to discharge: 38.67 days (17.19)* (ii) vIOP: 3 × 3 h virtual group session + 1 day per week attend medical and psychiatric appointments per week *Calendar days from treatment initiation to discharge: 50.83 (13.83)*	(i) PHP: In person & group + family (ii) vIOP: Virtual & group + family	NR	Yes (vital) To provide twice-weekly psychoeducational support groups focused on enhancing parental self-efficacy
*Programs for adults*								
Home-based treatments								
Eating Disorder and Nutrition Unit, CHU Le Bocage [Dijon, France] Daniel et al. [64]	Adults BN	(1) 5 BP episodes/week (2) BN duration > 2 years (3) Poor improvement (< 20% decrease in BP frequency) despite 3-month psychotherapy and/or use of antidepressant drug	(i) Tube feeding (ii) Tube feeding + CBT	No	*A period of 3 months* (mean duration of home-TF was 2.83 ± 2.28 months)	In person (nasogastric tube)	NR	NR
Community-Based Treatments								
Ladder to the Moon Program [Atlanta-Georgia, USA] Crenshaw [65]	Adults Any EDs	*Study inclusion criteria:* (1) Women 18 years old or older (2) Diagnosis of AN or BN according to the DSM-IV (3) Completion of the intensive outpatient program for EDs at Ladder to the Moon (4) Not currently a psychotherapy patient of the researcher	Feminist Consciousness	No	3 × 3-h group therapy sessions per week *A period of 3 weeks to 6 months with the average length of stay being 2 to 3 months*	In person & group	NR	NR
Amarum Expertise Centre for Eating Disorders [Netherlands] Deumens et al. [66], Lammers et al. [71], Vroling et al. [79], Lammers et al. [70], Lammers et al. [69]	Adults BED	Deumens et al. [66]; Lammers et al. [71]; Vroling et al. 2016: NR Lammers et al. [70]—Study inclusion criteria: (1) Patients with a BMI ≥ 30 (2) An above average urge to eat in response to negative emotions (score ≥ 2.38 on the DEBQ subscale Emotional Eating) Lammers et al. [69]—Study inclusion criteria: (1) Individuals with BED (DSM-5) or with subthreshold BED (those with BED of low frequency and those with subjective binge eating episodes)	(i) CBT + psychomotor therapy (ii) DBT-BED (only in Lammers et al. [70]; Lammers et al. [69])	No	(i) CBT + : 1-day group intervention per week containing 3 blocks (each 75 min) (ii) DBT-BED: 20 × 2-h group-sessions (only in Lammers et al. 2020; Lammers et al. 2022) *A period of 20 weeks*	In person & group	(i) CBT + : A psychologist, a psychiatric nurse and a psychomotor therapist (ii) DBT-BED: Trained psychologists/psychotherapists (only in Lammers et al. 2020; Lammers et al. 2022)	Yes (additional in CBT +) To offer six group meetings (90 min each) for patients and their partners, aimed at enhancing mutual understanding and support during the process of change
Anorexia Nervosa Intensive Treatment Team (ANITT) [Lothian region, UK] Munro et al. [77]; Hannon et al. [68]; Munro et al. [76]	Adults AN	(1) BMI < 13 kg/m2 OR > 15 kg/m2 and losing weight (more than 1 kg per week)	Schema Therapy	Yes	Contact varies from 2 to 10 contacts a week, depending on progress and the stage of treatment *A period of 18 months with 6 month period of reduced-intensity treatment (progress reviews)*	In-person & individual	Consultant psychiatrist in psychotherapy, consultant clinical psychologist, clinical psychologists, clinical associate in applied psychology, dieticians, nurse, assistant psychologists	NR
Behavioral Wellness Clinic/Louisville Center for Eating Disorders [Louisville, KY, USA] Levinson et al. [72]	Adults any EDs	(1) A diagnosis of an ED (2) Medical stability (determined via a medical provider) (3) BMI > 16.5 (4) Need for more intense care than outpatient alone	CBT-E + FBT + Exposure therapy + DBT	Yes	3 h daily for 5 days per week *Lengh of stay (weeks)* *(i) Telehealth: M(SD) = 11.07 (6.3)* *(ii) In-person: M(SD) = 12 (8.03)*	(i) In person group + individual (ii) Virtual group + individual	Therapist, dietitian, and prescriber	NR
Lui [73] [USA]	Adult BN, alcohol and cocaine use disorders, and bipolar I disorder	NR	individual: integrative cognitive affective therapy group: narrative therapy + ACT + DBT	Yes	15 × 1 h individual therapy + 3 × 3 h group therapy sessions per week Length NR	In person & individual + group	Therapist, dietician, psychiatrist	NR
Centre for Mental Health, University Health Network [Toronto, Canada] MacDonald et al. [75]	Adults Any EDs	*Study inclusion criteria:* (1) Had pre-IPT/DPT symptoms meeting criteria for DSM-5 diagnoses of: AN-R; AN-BP; BN; or PD (2) Achieved partial remission or better following IPT/DPT (3) Participated in ≥ 4 weeks of maintenance treatment (4) Participated in follow-up assessment at 6 and/or 12-months after IPT/ DPT	CBT	Yes	(i) IOP Group: 6 to 14 h per week *up to 16 weeks* (ii) Individual CBT: 16 sessions *over 14 weeks*	(i) In person & group (ii) In person & individual	NR	NR
Centre for Mental Health, University Health Network [Toronto, Canada] MacDonald et al. [74]	Adults ARFID	Eligible participants: (1) Had a DSM-5 diagnosis of ARFID (2) Started treatment at the University Health Network’s Eating Disorder Program between April 2020 and March 2023	CBT-AR + CBT + DBT	Yes	CBT-AR: 2 × per week for the first 16 sessions and 1 × per week for the final 4 sessions Group: one clinician-supported meal and one psychotherapy group per day, from Monday to Friday *A period of 12 weeks*	In person & individual + group	A multidisciplinary care team (i.e., psychologists, psychiatrists, nurse practitioners and nurses, social workers, registered dietitians, occupational therapists, and registered psychotherapists)	NR
Yorkshire Centre for Eating Disorders [Leeds, UK] Saeidi et al. [78]	Adults AN	*Study inclusion criteria*—participants had to meet at least two of the following:(1) DSM-IV diagnostic criteria for AN for minimum of four years (2) BMI of 12 and above (3) Several admissions to specialist EDs services (4) Lack of response to long-term individual or group therapy (5) Aged 18 years and above	NR	NR	2 to 7.5 h per week, up to five visits per week *Length NR*	In-person, telephone or text	Health support workers, dietitian, consultant psychiatrist, nurse	NR
Bright Heart Health [23 states in USA] Wilkes [80]	Adults Any EDs	*Study inclusion* (1) Willingness to provide demographic and insurance information (2) Willingness to complete various assessment (3) Commitment to pay for services and participate (4) Access to internet (5) Ongoing medical assessments with consent to release information to treatment team	CBT + DBT + experiential therapy somatic therapy	Yes (where patients eat a meal online once a week and psychoeducation on meal planning and nutrition)	11-h per week *Average period of 10 weeks*	Virtual & individual + group	Marriage and family therapists, clinical social workers, psychologists, dieticians, and a psychiatrist	Yes To provide family sessions and support groups, equipping family members with the tools to support their loved ones during treatment
Cleveland Center for Eating Disorders [Cleveland—Ohio, USA] Federici et al. [67]	Adults Any EDs	(1) Experienced repeated treatment failures from standard day treatment, residential, and/or inpatient settings *OR* participated in standard EDs programming for a minimum of 28 days without a decrease in symptoms (2) One or more of the following criteria: (i) Presenting as Mult diagnostic, as evidenced by an additional co-occurring Axis I disorder/s and/or Axis II disorder/s (ii) Struggle with pervasive emotion regulation deficits that commonly lead to symptoms, as evidenced by an inability to adaptively regulate, communicate, or tolerate affect (iii) Historically have been unable to generalize skills outside of standard treatment, as evidenced by relapse in symptoms posttreatment or during treatment (iv) Present with considerable therapy-interfering behavior(s) such that they cannot remain in standard treatment for eating disorders without significant adverse consequences to the therapy milieu	DBT + CBT components	Yes (meal planning)	DPT: 6 h per day for 5 days/week or IOP: 3 h per day for 3–5 days/week *A period of 6 months*	In person & individual + group	DBT therapist, nutritionists and psychiatrists	NR
*Programs for all age*								
Home-based treatments								
Hellenic Center for Eating Disorders [Nea Kifisia, Greece] Tsiaka and Bletsos [81]	Adolescents and adults AN	(1) 13 + years old (2) Low weight, need for refeeding and meal support BMI < 15 BMI > 15 medically stable or with unstable biochemistry (3) Meet the diagnostic criteria for severe or enduring AN and related conditions (4) Prescribed specialist treatment recommended to reduce risk of relapse and move to discharge from medical services	MANTRA + Cognitive Interpersonal Maintenance Model	Yes (providing a model for healthy eating, meal plan, cooking sessions)	4–6 h per day for everyday *A period of 18 weeks*	In person & family	Medical Doctors: Pathologist, Cardiologist, Endocrinologist, Pediatrician Treatment Team: Psychiatrist, psychologists, family therapists, nutritionist/dietician, occupational therapist, psychiatric nurse	Yes (vital) To provide staff-supported family meals To provide family therapy
Community-based treatments								
Eating Recovery Center [USA] Blalock et al. [82]; Rienecke et al. [84]	Children, adolescents and adults Any EDs	Blalock et al. [82]: (1) Qualify for IOP level of care (2) Have insurance (patients were not charged for VIOP, but were required to have insurance in case a step up to higher level of care was needed) (3) Have a reliable internet connection at home as well as access to a computer, web camera, and headphones (4) Receive medical clearance from an in-person local medical provider Rienecke et al. [84]: NR	DBT + CBT + ACT For C&A service: + EFFT	Yes (meal support group -feedback over video from therapists or dietitians on portions and nutrition + eating meals together with therapists or dietitians and peers)	3 × 3-h group therapy sessions weekly, 1 h of individual or family therapy weekly, one biweekly appointment with a registered dietitian Blalock et al. [82]: A biweekly in-person meeting with the patient's local medical provider Rienecke et al. [84]: Medical monitoring collected by remote medical devices Blalock et al. [82]: *A period of 6 weeks with an average of M (SD) = 5.85 (1.72) weeks* Rienecke et al. [84]: *The average number of days treatment for adults was 62.41 days (SD* = *29.90) and for children/adolescents was 79.48 days (SD* = *37.04)*	Blalock et al. [82]: In person & virtual & group + individual or family Rienecke et al. [84]: Virtual & group + individual or family	A licensed psychotherapist, a registered dietitian and a consulting psychiatrist (on-hand for support)	Yes To replace individual therapy with family therapy when clinically indicated, focusing on meal planning and problem-solving To offer optional emotion-focused family therapy EFFT training sessions for parents or guardians
Rodríguez Guarin et al. [85]	Adolescents and adults Any EDs	Absence of medical or psychiatric life-threatening risk	CBT + Psychodynamic psychotherapy + DBT + Expressive therapy + Art therapy	Yes (nutritional rehabilitation, daily therapeutic meals)	~ 19 h per week (2 individual sessions + 1 Psychiatry follow-up session + 2 daily group sessions + 1 weekly nutrition workshop + multi-family and single-family support sessions) *Length not clear – at least 8 weeks*	Virtual & individual + group	Psychiatrists, psychologists and two healthcare professionals working in nutrition	Yes To train and to support parents and caregivers (e.g., modelling for how to proceed at mealtimes)
[Northeastern USA] Walker et al. [86]	Adolescents and adults Any EDs	NR	CBT-E + DBT	Yes	4-h per day, 3 days per week from 2013 to 2015 and up to 4 days per week from 2015 to 2017 *Average of 15.82 (SD* = *13.38) weeks*	In person & group	A licensed masters or doctoral level clinician or by a graduate student under the direct supervision of a licensed clinician (not specified)	NR
The Renfrew Center [USA] Lowe et al. [83]	Adolescents and adults Any EDs	NR	CBT	Yes	3 × 3 h per week *Length NR*	In person & individual + group	Case manager, registered dietitian, psychiatrist, master's level clinicians—not specified)	Yes (optional) family therapy—details not specified
Within Health [USA] Wolfe et al. [87]	Adolescents and adults Any EDs	NR	Integrative treatment model: ACT, CBT, DBT, interpersonal with experiential modalities (e.g., art therapy, movement) (indicated based on patient presentation)	Yes (meal support group + meal planning check-ins + nutrition counseling)	At least 3 h per day; 3–5 days per week *Length NR*	Virtual & individual + group	A multidisciplinary team of professionals, including a psychotherapist, registered dietitian, registered nurse, psychiatric provider, and clinical support staff (e.g., care partner, food specialist)	Yes (where indicated) To address couples/family-related issues including increasing understanding of EDs, working on communication, and cultivating supportive relationships

ACT: acceptance and commitment therapy; AN: anorexia nervosa; AN-BP: anorexia nervosa, binge-purge type; AN-R: anorexia nervosa, restricting type; ARFID: avoidant/restrictive food intake disorder; BED: binge eating disorder; BMI: body mass index; BN: bulimia nervosa; CAMHS: Child and adolescent mental health services; C&A: children and adolescents; CBT: cognitive behavioral therapy; CBT-AR: cognitive-behavioral therapy for avoidant/restrictive food intake; CBT-E: enhanced cognitive behavioral therapy; CRT: cognitive remediation therapy; DBT: dialectical behavior therapy; DEBQ: Dutch eating behavior questionnaire; DPT: day patient treatment; DSM: Diagnostic and Statistical Manual of Mental Disorders; ED: eating disorders; EDNOS: eating disorder not otherwise specified; EDIP: Eating Disorder Intensive Pathway; EFFT: emotion-focused family therapy; FBT: family based treatment; FT-ED: family therapy for eating disorders; HT: home treatment; ICD: International Classification of Diseases; IOP: intensive outpatient program; IPT: inpatient treatment; MANTRA: Maudsley anorexia nervosa treatment for adults; MBSR: mindfulness-based stress reduction; M (SD): mean(standard deviation); NR: not reported; PHP: partial hospitalization; TAU: treatment as usual; UK: United Kingdom; USA: United States of America; VIOP: virtual intensive outpatient program

#### Programs for children and adolescents

##### Home-based treatments

Seven HBT programs for children and adolescents were identified which were presented in 13 distinct papers [15, 42–54]. These HBT programs were predominantly designed to treat AN and atypical AN, except two providing treatment/care for any type of ED [44, 53]. Family-Based Treatment (FBT) was the most common psychotherapeutic approach, either as a stand-alone treatment or combined with Cognitive Behavior Therapy (CBT). In all HBT programs, family/carer involvement was reported as a vital part of treatment, mainly focusing on providing psychoeducation about EDs and guidance on managing eating behaviors and stress. Furthermore, these programs provided meal support and/or supervised meals, except one program [49, 50] that offered supervised meals during the initial inpatient admission period, but for which the provision of such support during the home treatment phase was not clearly indicated.

All treatments were designed to include visits to patients’ homes, yet one case study described virtual treatment due to the COVID-19 pandemic [51]. While the treatments were delivered by multidisciplinary teams in most of the HBT programs, two programs used nurses as the main healthcare professionals delivering the treatment [44, 45, 52, 54].

The duration and intensity of HBT programs appeared to vary, mostly depending on clinical severity and healthcare systems. The minimum duration was six weeks [44] and the maximum duration was reported as 16 weeks with an option to go up to 32 weeks based on available insurance coverage [47, 48]. Contact intensity varied significantly across different programs with the most intensive ones providing daily home visits (Monday–Friday) of 30–60 min each [53] or up to 10 home visits per week (Monday- to Friday; [44]. The least intensive treatment included one to four sessions per week each lasting for approximately 60 min, with an average of 1.8 sessions [45, 52, 54].

Information on HBT admission criteria was not always clearly presented. Several papers listed “inclusion criteria” for the research component instead of precisely outlining admission criteria for HBT programs. Among the specified eligibility and/or inclusion criteria, availability of a caregiver for involvement in treatment and distance to the hospital (e.g., less than 30 min commute from home to hospital) were common requirements.

##### Intensive community treatments

Four ICT programs for children and adolescents were identified, which were presented in nine papers [55–63]. These offered care for any type of ED. However, one of these programs, also offered HBT-support and outreach when deemed necessary [59, 60]. Treatments involved combinations of psychotherapeutic approaches such as FBT, CBT and Dialectical Behavior Therapy (DBT) as well as meal support and/or supervised meals, except for one virtual program [63] which did not offer meal support. Family/carer involvement was a vital component across the programs with a focus on providing parents with psychoeducation about EDs, guidance on managing eating behaviors and empowering them.

Of these programs, only one delivered treatment in-person [55–58, 61]. One program offered virtual treatment [63], and two had a hybrid format [59, 60, 62]. Furthermore, while two programs were delivered by multidisciplinary teams [59, 60, 62], two did not specify the healthcare professionals involved [55–58, 61, 63]. The duration of ICT programs for this age group were mostly around eight weeks, and the intensity ranged from three-four hours/week [62] to 10 h/week [63]. Other than being under 18 years old and having an ED diagnosis, there were no common eligibility or inclusion criteria.

Overall, neither the discharge criteria nor the post-treatment procedure were explicitly outlined for any of the ICT or HBT programs for children and adolescents.

#### Programs for adults

##### Home-based treatments

Only one HBT program for adults was identified: this focused on bulimia nervosa (BN) [64]. The treatment involved tube feeding delivered at home for a period of 3 months. This treatment program did not provide information regarding the involvement of professionals delivering the intervention, nor the involvement of families or caregivers.

##### Intensive community treatments

ICT programs for adults were more diverse, with 10 programs offering treatment presented in 16 distinctive sources [65–80]. The majority of these (n = 5) provided treatment for patients with any type of ED. Two programs offered treatment for AN only [68, 76–78], one for binge eating disorder (BED) [66, 69–71, 79], one for BN [73], and one for avoidant/restrictive food intake disorder (ARFID) [74].

ICT programs for adults primarily offered a combination of psychotherapeutic approaches, such as CBT and DBT. One program used on Schema Therapy [68, 76, 77], while another used a ‘Feminist Consciousness’ approach [65]. The majority of these programs provided meal support and/or supervised meals, except for three programs [65, 66, 69–71, 78, 79]. Only one program had family/carer involvement as a core component, providing tools to help family members support their loved one during treatment [80]. Another program offered family involvement as an additional component with the goal of improving understanding and support amongst parents and partners during the process of change [66, 69–71, 79].

These programs offered treatment in-person, except for two virtual programs [72, 80]. Multidisciplinary treatment teams commonly delivered these programs, although two programs did not report this information [31, 65]. Regarding the duration of programs, the Anorexia Nervosa Intensive Treatment Team (ANITT) in the UK was the longest with a duration of up to 18 months and an additional 6-month period of reduced-intensity treatment. This program focused on very severe and persistent AN only [68, 76, 77]. The shortest reported treatment duration was 3 weeks, with the average treatment duration typically ranging from 2 to 3 months [65].The treatment programs typically involved several hours per week, combining group and individual sessions. Contact hours ranged from three 75-min blocks of a weekly group intervention all delivered on one day [66, 69–71, 79] to 15 h per week [72].

The eligibility and/or study inclusion criteria for these treatment programs were diverse, reflecting a range of approaches and criteria. Commonly reported criteria included diagnostic aspects and specific body mass index (BMI) levels. Discharge criteria were mentioned in only one ICT program [80] as follows: no ED behaviors for two weeks and completing 90% of the meal plan at least 90% of the time.

#### Programs for all age populations

##### Home-based treatments

One was a HBT program providing care for adolescents and adults with AN [81]. This program offered The Maudsley Anorexia Nervosa Treatment for Adults (MANTRA) as its main psychotherapeutic modality combined with meal support and/or supervised meals daily over a period of 18 weeks. Family/carer involvement was seen as integral to this program, with a focus on family therapy and supporting families during mealtimes. Treatment was delivered by a multidisciplinary team.

##### Intensive community treatments

Five ICT programs provided treatment for any type of ED for all age populations [82–87]. These programs offered meal support and/or supervised meals. While one employed CBT as a stand-alone approach [83], others used CBT combined with other psychotherapeutic approaches such as DBT or Acceptance and Commitment Therapy (ACT) [82, 84–87]. While family/carer involvement was offered in three programs as needed [82–84, 87], itwas a main component in one program [85] and was not reported in another [86]. Four programs described the treatment team as involving professionals from various backgrounds (e.g., psychiatrist, dietitian) [82–85, 87], whereas one paper did not specify the professional roles beyond licensed clinicians or supervised graduate students [86]. The duration of treatment was not reported for two program [83, 87] and it was not clear for one (i.e., at least eight weeks; [85]), while two other programs reported an average duration of about 16 weeks [82, 84, 86]. The weekly intensity was similar across programs, ranging from nine to 19 h/week.

Admission criteria were only specified for the HBT program [81] and in one paper describing an ICT program [82]. Discharge criteria were not mentioned for any of the programs.

### Efficacy, acceptability and cost-effectiveness of the treatment programs

Table 4 presents a summary of the quantitative outcomes and Table 5 provides a summary of the qualitative outcomes. To map the types of evidence and study designs, the included papers were classified according to the JBI Levels of Evidence classification system which is a hierarchical framework that ranks quality of evidence from Level 1 (strongest) to Level 5 (weakest) based on study design and methodological rigor.

**Table 4:** Summary of quantitative outcomes: ED-related outcomes, feasibility and acceptability, and cost-effectiveness

Treatment programs and author(s)	N	Design	Distribution of participants by diagnosis	Brief treatment description	EDs related outcomes (e.g., BMI, symptoms)	Feasibility and acceptability outcomes	Cost-effectiveness outcomes
*Programs for children and adolescents*							
Home-based treatments							
Department of Child and Adolescent Psychiatry, University of Zürich [Zürich, Switzerland] Flütsch et al. [45]	45	Case Series/Pilot Study	AN: n = 31 (69%) AN atypical: n = 14 (31%)	FBT at home 1–4 × 1 h sessions per week + additional regular OP *A period of 12 weeks*	BMI: A significant improvement with a large effect size EDE: A significant improvement with a medium effect size EDI-2: A significant improvement with a large effect size	(1) Good treatment retention with no premature dropouts during the study period (2) Treatment was well accepted by the majority of patients and parents, rating their treatment satisfaction as good or very good	NR
Department of Child and Adolescent Psychiatry, University of Zürich [Zürich, Switzerland] Pauli et al. [54]	HBT + FBT: 45 FBT only: 22	Waiting List Control Design Pilot Study	AN: n = 52 (77.6%) AN atypical: n = 15 (22.4%)	(i) FBT only: at least 1 × 1 h per week (ii) HBT + FBT: 1–4 × 1 h sessions per week + additional regular OP *A period of 12 weeks*	BMI: A significantly higher increase in the HT + FBT group compared to the FBT‐only group 3 months after the beginning of treatment EDE: Both groups showed a significant improvement from pre treatment to 3 months after the beginning of treatment without a significant group difference EDI‐2: Both groups showed a significant improvement from pre treatment to 3 months after the beginning of treatment without a significant group difference Over‐exercising: Both groups showed a significant improvement from pre treatment to 3 months after the beginning of treatment without a significant group difference	No patients in either group dropped out of the treatment and the study during the study period	NR
Department of Child and Adolescent Psychiatry, University of Zürich [Zürich, Switzerland] Mayr et al. [52]*	HBT + FBT: 40 FBT only: 21		AN: n = 52 (85.2%) AN atypical: 9 (14.8%)	Same as Pauli et al. [54]	EBW: In the HBT + FBT group 70% of patients reached > 85% of their EBW within 3 months, whereas in the FBT‐only group this was achieved by only 52% of patients		The average cost per subject in the HBT + FBT group was 5770 SFr. (Swiss francs), nearly half the cost of 10,710 SFr. per subject in the FBT‐only group Inpatient treatment comprised 86% of the total cost faced by the FBT‐only group
Child and Adolescent Home Treatment Team [Gloucestershire, UK] Clark-Stone et al. [44]	33	Service Evaluation	AN: 87.88% (n = 29) BN: 6.06% (n = 2) OSFED: 6.06% (n = 2)	FBT Starting with 10 contacts per week by gradually reducing *A period of 6 weeks*	Of the 31 patients, 70.97% of them had an increased BMI compared to 29.03% who had BMI decreases	NR	NR
Department of Child and Adolescent Psychiatry, Psychosomatics and Psychotherapy of the RWTH Aachen [Aachen, Germany] Herpertz-Dahlmann et al. [50]	Patients: 22 Carers: 22	Single-centre, non-randomized, open-label pilot study	AN: 100% AN atypical: n = 3 (13.6%)	FBT + CBT Starting 3–4 × visits per week (1st and 2nd month), then 1–2 × visit per week (3rd and 4th month) *A period of 4 months*	BMI: Significant improvement at the end of HT and 1-year follow-up Weight: Highest weight gain was achieved between IP admission and beginning of HT. Weight continued to increase during HT, and between the end of HT and the 1-year follow-up, weight gain was maintained EDI-2: Significant improvement from beginning of HT to end of HT. This improvement was maintained for the 1-year follow-up	Both patients and carers reported high treatment satisfaction	HT reduced cost around 25%
Department of Child and Adolescent Psychiatry, Psychosomatics and Psychotherapy of the RWTH Aachen [Aachen, Germany] Heider et al. [49]	21	Single-centre, non-randomized, open-label pilot study	AN: n = 18 (85.7%) AN atypical: n = 3 (14.3%)	FBT + CBT Starting 3–4 × visits per week (1st and 2nd month), then 1–2 × visit per week (3rd and 4th month) *A period of 4 months*	Same as Herpertz-Dahlmann et al. 2020	Same as Herpertz-Dahlmann et al. 2020	Same as Herpertz-Dahlmann et al. 2020
Hospital Infantil Universitario Niño Jesús [Madrid, Spain] Morón-Nozaleda et al. [53]	Patients: 57 Carers: 43	Retrospective Feasibility Study	AN (restrictive type): n = 30 (50.8%) AN atypical: n = 7 (11.8%) AN (purging type): n = 3 (5.1%) BN: n = 5 (8.5%) ARFID: n = 3 (5.1%) OSFED: n = 11 (18.6%)	CBT + FBT Daily home visits from Monday to Friday approximately 30–60 min + 2 programmed calls per day *Mean stay of 39.14 days (SD* = *14.47)*	NR	(1) 91.5% of the patients complied with the complete protocol (2) The overall family satisfaction was high (3) All caregivers perceived the program as “very safe.”	In its first year of operation, the program avoided a total of 2016.03 inpatient hospital stays (saving a total of 1,762,292.46 euros compared to conventional inpatient treatment)
Community-Based Treatments							
The Walden Adolescent Intensive Outpatient Program [MA, USA] Chiumiento [55]	272	Secondary Analyses of Archival Data	AN: n = 117 (41.2%) EDNOS: n = 155 (54.6%) BN: n = 8 (2.8%) ARFID: n = 4 (1.4%)	Parent DBT Skills Group vs Parent Support Group vs Multi-Family Group 1 × per week *A period of 8 weeks, or 24-day period*	NR	No significant differences between satisfaction rating of program components (DBT parenting skills group, parent support group, MFT group)	NR
The Walden Adolescent Intensive Outpatient Program [MA, USA] Doyle [56]	Children: 14 Adolescents: 30	Secondary Analyses of Archival Data	AN: n = 15 (34%) EDNOS: n = 28 (64%) BN: n = 1 (2.2%)	FBT(Maudsley Approach) + CBT + DBT 3 × 3-h group therapy sessions per week *A period of 8 weeks, or 24-day period*	BMI/IBW: Both age groups showed significant improvements from pre- to post-treatment and to 6-month follow-up EDE-Q total: Both age groups showed significant improvements from pre- to post-treatment and to 6-month follow-up	NR	NR
The Walden Adolescent Intensive Outpatient Program [MA, USA] Johston et al. [57]	51	Pilot study	AN: n = 17 (33%) BN: n = 6 (12%) EDNOS: n = 28 (55%)	FBT(Maudsley Approach) + CBT + DBT 3 × 3-h group therapy sessions per week *A period of 8 weeks, or 24-day period*	BMI: Statistically significant increase from admission to discharge, discharge to 3 month follow-up, and 6 month and 1 year follow-ups EDE-Q: Statistically significant improvement from admission to discharge, and continued to improve at 6 months and were similar at 1 year follow-up Binge–purge behaviors: Frequency decreased over the course of treatment, but not significantly	(1) 36/51 patients (71%) completed the full program (2) Overall treatment attrition rate was 29%	NR
The Walden Adolescent Intensive Outpatient Program[MA, USA] Kim [58]	36	Secondary Analyses of Archival Data	AN: n = 18 (50%) BN: n = 1 (2.8%) EDNOS: n = 17 (47.2%)	FBT(Maudsley Approach) + CBT + DBT 3 × 3-h group therapy sessions per week *A period of 8 weeks, or 24-day period*	BMI/IBW: Statistically significant improvement from admission to discharge, and continued to increase at 3 months to 6-month follow-ups Binge–purge behaviors: No statistically significant difference EDE-Q total: Statistically significant improvement from admission to discharge, and continued to improve from discharge to 1 year follow-up	NR	NR
Eating Disorder Intensive Pathway—East London Foundation Trust [London, UK] Kuang et al. [60]	32	Service Evaluation	AN: n = 25 (78.1%) OSFED: n = 6 (18.8%) BN: n = 1 (3.1%)	FT-ED (Maudsley Approach) + distress tolerance + CBT An average of 5 h per week; a maximum of 24 contacts per week *A period of 6 to 8 weeks*	W4H: Statistically significant improvement from pre- to post-treatment CGAS: Statistically significant improvement from pre- to post-treatment EDE-Q: No statistically significant difference	NR	NR
The Walden Adolescent Intensive Outpatient Program [MA, USA] Monk [61]	137	Secondary Analyses of Archival Data	AN: n = 52 (38.2%) EDNOS: n = 82 (60.3%) BN: n = 2 (1.5%)	FBT (Maudsley Approach) + CBT + DBT 3 × 3-h group therapy sessions per week *A period of 8 weeks, or 24-day period*	NR	NR	NR
Michigan Medicine Comprehensive Eating Disorders Program [Michigan, USA] Van Huysse et al. [63]	(i) PHP: 49 (ii) vIOP: 53	Naturalistic Cohort Study	(i) In-person PHP AN (restrictive type): n = 26 (53.1%) AN (binge-purge type): n = 4 (8.2%) BN: n = 4 (8.2%) ARFID: n = 4 (8.2%) OSFED-atypical AN: n = 11 (22.4%) OSFED-other: 0 (0%) (ii) Virtual IOP AN (restrictive type): n = 31 (58.5%) AN (binge-purge type): n = 5 (9.4%) BN: n = 1 (1.9%) ARFID: n = 1 (1.9%) OSFED-atypical AN: n = 13 (24.5%) OSFED-other: n = 2 (3.8%)	FBT (primary) + CBT + DBT (i) PHP: 6 h per day for a 5 days a week (~ 32 h over 5 days weekly) *Calendar days from treatment initiation to discharge: 38.67 days (17.19)* (ii) vIOP: 3 × 3 h virtual group session + 1 day patients attend medical and psychiatric appointments per week (~ 13 h over 4 days weekly *Calendar days from treatment initiation to discharge: 50.83 (13.83)*	Expected body weight (%EBW): In both groups, patients showed significant improvements in %EBW over time, with average %EBWs suggesting that participants were at or very close to weight restored at 3- and 6-months post-treatment	NR	Amount billed was $30,296 for vIOP and $64,854 for PHP
*Programs for adults*							
Home-based treatments							
Eating Disorder and Nutrition Unit, CHU Le Bocage [Dijon, France] Daniel et al. [64]	118 (i) Tube feeding (TF) + CBT: 61 patients (ii) Tube feeding (TF) alone: 57 patients	Open Prospective Study	BN: 100%	(i) Tube feeding (ii) Tube feeding + CBT	The frequency of binge-purge episodes was lower after treatment The improvement in energy and protein intake was similar in the two groups	Home-tube feeding was quoted as too hard in 12% of the patients who stopped it, very hard (very difficult) in 24% of them, hard in 35%, easy in 24% and very easy in 5% of them	NR
Community-Based Treatments							
Eating Recovery Center [USA] Blalock et al. [82]	57	Naturalistic Pilot Feasibility Study	AN: n = 21 (37%) BN: n = 21 (37%) BED: n = 9 (16%) ARFID: n = 3 (5%) OSFED: n = 3 (5%)	DBT + CBT + ACT 3 × 3-h group therapy sessions weekly, 1 h of individual or family therapy weekly, one biweekly appointment with a registered dietitian and a biweekly in-person meeting with the patient's local medical provider A period of 6 weeks with an average of M (SD) = 5.85 (1.72) weeks	Binging: statistically significant and clinically meaningful improvements Purging: statistically significant and clinically meaningful improvements Restricting: statistically significant and clinically meaningful improvements	(1) All patients attended all therapy during treatment period (2) Patients strongly endorsed VIOP as a helpful experience	NR
Amarum Expertise Centre for Eating Disorders [Netherlands] Deumens et al. [66]	182*	Naturalistic Study	BED: 100%	1-day group intervention per week containing 3 blocks (each 75 min) *A period of 20 weeks*	BMI: Statistically significant improvements EDI-II Drive for thinness: Statistically significant improvement EDI-II Interceptive awareness: Statistically significant improvement EDI-II Bulimia: Statistically significant improvement Body attitude: Statistically significant improvement	NR	NR
Amarum Expertise Centre for Eating Disorders [Netherlands] Lammers et al. [71]	431	Naturalistic Study	BED: 100%	1-day group intervention per week containing 3 blocks (each 75 min) *A period of 20 weeks*	BMI: Statistically significant improvement from baseline to end of treatment (moderate effect size) and from end of treatment to 6 month follow up (weak effect size) EDI-II Bulimia: Statistically significant improvement (large effect size) from baseline to end of treatment	90 patients dropped out of treatment (83 women and 7 men)	NR
Amarum Expertise Centre for Eating Disorders[ Netherlands] Vroling et al. [79]	376	Naturalistic Cohort Study (secondary analysis)	BED: 100%	1-day group intervention per week containing 3 blocks (each 75 min) *A period of 20 weeks*	NR	21.81% of the patients dropped out of treatment	NR
Amarum Expertise Centre for Eating Disorders [Netherlands] Lammers et al. [70]	(i) CBT + : n = 33 (ii) DBT-BED: n = 41	Open, Quasi-randomized, Controlled trial	BED: 100%	(i) CBT + : 1-day group intervention per week containing 3 blocks (each 75 min) (ii) DBT-BED: 20 × 2-h group-sessions *A period of 20 weeks*	EDE-Q Global: The CBT + group experienced greater reductions that approached significance at end of treatment and reached significance at 6 month follow-up Objective binge eating episodes: The CBT + group showed greater reductions at end of treatment, but these differences were no longer significant at 6 month follow-up Emotional eating: No significant group differences	7 (9.5%) participants dropped out of the treatment and/or study during the course of the trial, including 2 (6.1%) from CBT + and 5 (12.2%) from DBT-BED	NR
Amarum Expertise Centre for Eating Disorders [Netherlands] Lammers et al. [69]	(i) CBT + : n = 133 (ii) DBT-BED: n = 42	Open, Quasi-randomized, Controlled trial	BED: 100%	(i) CBT + : 1-day group intervention per week containing 3 blocks (each 75 min) (ii) DBT-BED: 20 × 2-h group-sessions *A period of 20 weeks*	EDE-Q Global: Both groups showed statistically significant improvement between baseline and EOT and between baseline and follow-up, but improvement was greater in CBT + group (medium effect size) between baseline and EOT Objective binge eating episodes: Scores in both groups decreased significantly between baseline and EOT, and between baseline and 6-month follow-up Emotional eating: CBT + group (small effect size) showed statistically significant improvement between baseline and EOT	28 (16.0%) participants dropped out of the treatment during the course of the trial, including 20 (15.0%) from CBT + and 8 (19.0%) from DBT-BED	NR
Anorexia Nervosa Intensive Treatment Team (ANITT) [Lothian region, UK] Munro et al. [77]	33	Service Evaluation	AN: 100%	Schema Therapy 2 to 10 contacts a week *A period of 18-month with 6-month period of reduced-intensity treatment (progress reviews)*	NR	(1) The mean overall service satisfaction was 4/5 (2) Patients perceived staff as supportive, caring and genuine (3) Patients valued individualised care, a holistic psychological approach based on emotional and physical needs and not just weight (4) Only 2 patients dropped out during a 2 year period	A total saving of £391,656 in a 2 year period
Anorexia Nervosa Intensive Treatment Team (ANITT) [Lothian region, UK] Munro et al. [76]	26	Naturalistic Case Series	AN: n = 24 (92%) Atypical AN: n = 2 (8%)	Schema Therapy 2 to 10 contacts a week *A period of 18-month with 6-month period of reduced-intensity treatment (progress reviews)*	BMI: Increased on average by 3.9 kg/m2 from a mean of 13.0 at intake to 16.9 at the end of the study period EAT-26: 7 patients (27%) showed statistically significant change, with five (19%) achieving change to the level of remission or recovery. 13 patients (50%) showed no statistically significant change, with nine of these showing non-significant improvement. Six patients (23%) described statistically significant deterioration	75% of the sample were either ‘satisfied’ or ‘extremely satisfied’ with the treatment	NR
Behavioral Wellness Clinic/Louisville Center for Eating Disorders [Louisville, KY, USA] Levinson et al. [72]	(i) In-person: 60 patients (ii) Telehealth: 33 patients	Nonrandomized Control Trial	(i) In-person AN: n = 26 (43.33%) BN: n = 6 (10.00%) OSFED: n = 22 (26.67%) BED: n = 5 (8.33%) ARFID: n = 1 (1.67%) (ii) Telehealth AN: n = 14 (42.4%) BN: n = 4 (12.12%) OSFED: n = 10 (30.03%) BED: n = 4 (12.12%) ARFID: n = 1 (3.03%)	CBT-E + FBT + Exposure therapy + DBT 3 h daily for 5 days per week *Length of stay (weeks)* *(i) Telehealth: M(SD)* = *11.07 (6.3)* *(ii) In-person: M(SD)* = *12 (8.03)*	BMI: Both groups had significant improvement at discharge EDE-Q: Both groups had significant improvement at discharge	NR	NR
Lui [73] [USA]	1	Case study	BN: 100%	Individual: integrative cognitive affective therapy & group: narrative therapy + ACT + DBT 15 × 1 h individual therapy + 3 × 3-h group therapy sessions per week Length NR	BMI: No change EDE-Q: Patients scores reduced from baseline to end of treatment, and from end of treatment to 1-month follow-up EPSI: Patients scores reduced from baseline to end of treatment, and from end of treatment to 1-month follow-up for body dissatisfaction, binge eating and negative attitudes toward obesity. Patient's cognitive constraint, purging and restriction scores reduced from baseline to end of treatment. Patient's excessive exercise score increased from baseline to end of treatment, but was lower at 1-month follow up compared to baseline	NR	NR
Centre for Mental Health, University Health Network [Toronto, Canada] MacDonald et al. [75]	(i) IOP Group: 103 patients (46.6%) (ii) Individual CBT: 118 patients (53.4%)	Sequential Cohort Study	(i) IOP Group:AN (restrictive type): 8.7% AN (binge-purge type): 9.7% BN: 63.1% PD: 18.4% (ii) Individual CBT Group: AN (restrictive type): 9.3% AN (binge-purge type): 10.2% BN: 66.9% PD: 13.6%	CBT (i) IOP Group: 6 to 14 h per week *up to 16 weeks* (ii) Individual CBT: 16 sessions *over 14 weeks*	BMI: AN patients in both treatment group showed an improvement ED symptoms: Both groups followed very similar trajectory of return to clinically significant symptoms at 12-month follow-up	NR	NR
Centre for Mental Health, University Health Network [Toronto, Canada] MacDonald et al. [74]	(i) IPT + IOP: 9 patients (ii) IPT + individual therapy: 6 patients (iii) IOP alone: 3 patients (iii) Individual therapy alone: 12 patients	Retrospective Chart Review	ARFID: 100% Initial assessment: n = 42 From IP to IOP: n = 8 Directly IOP: n = 3	CBT-AR + CBT + DBT CBT-AR: 2 × per week for the first 16 sessions and 1 × per week for the final 4 sessions Group: 1 clinician-supported meal and 1 psychotherapy group every day	BMI: Patients showed improvement (only reported for those transitioned from IP to IOP)	(1) Of the nine patients who transitioned from inpatient to IOP, eight (88.9%) completed IOP and one (11.1%) ended treatment early (2) Three patients (7.1% of the total sample) had a direct entry to IOP, all of whom (100%) completed treatment	NR
Yorkshire Centre for Eating Disorders [Leeds, UK] Saeidi et al. [78]	Patients: (n = 6) Clinical team members (n = 8)	Service Evaluation	AN: 100%	2 to 7.5 h per week, up to five visits per week *Length NR*	Eating Disorders Quality of Life Scale: The average score for quality of life increased from 2 to 2.3, with social interaction the most improved area	Please qualitative findings regarding treatment experiences in Table 2	(1) Treatment reduced costs by 30% over 2 years as a result of reductions in the use of psychiatric hospital beds (2) The service reduced the number of inpatient admissions by 38%
Bright Heart Health [23 states of USA] Wilkes [80]	34	Naturalistic Feasibility Study	AN: n = 5 (14.7%) BN: n = 12 (35.3%) BED: n = 3 (8.8%) Atypical AN: n = 4 (11.8%) Low-frequency BED: n = 1 (2.9%) Night eating syndrome: n = 1 (2.9%) Other: n = 8 (22.9%)	CBT + DBT + experiential therapy + somatic therapy 11-h per week *Average period of 10 weeks*	BMI: Small to moderate improvement EDE-Q: Restraint, eating and weight concern subscales yielded moderate to large effect sizes, the shape concern and global score subscales yielded large effect sizes from baseline to end of treatment Binge/Purge: Significant improvements in frequency of binge eating, vomiting, laxative use, and compulsive exercise from baseline to end of treatment (small to moderate effect sizes)	(1) Treatment was proved to be moderately feasible (2) 56% of participants who started the program successfully completed the program while 15% of them dropped out (3) Majority of participants were satisfied with the service (medium to high level satisfaction) and quality of service and would recommend the program to a friend	NR
*Programs for all age*							
Community-based treatments							
Eating Recovery Center [USA] Rienecke et al. [84]	(i) Adults: 305 (ii) Children and adolescents: 33	Naturalistic Study	(i) Adults: AN-R (n, %) = 41 (13.4%) AN-BP (n, %) = 16 (5.2%) BN (n, %) = 21 (6.9%) BED (n, %) = 67 (22.0%) ARFID (n, %) = 9 (3.0%) OSFED (n, %) = 139 (45.6%) (ii) Children and adolescents: AN-R (n, %) = 11 (33.3%) AN-BP (n, %) = 3 (9.1%) BN (n, %) = 2 (6.1%) BED (n, %) = 2 (6.1%) ARFID (n, %) = 1 (3.0%) OSFED (n, %) = 14 (42.1%)	DBT + CBT + ACT + additional EFFT for C&A 3 × 3-h group therapy sessions weekly, 1 h of individual or family therapy weekly, one biweekly appointment with a registered dietitian *average number of days treatment for adults was 62.41 days (SD* = *29.90) and for children/adolescents was 79.48 days (SD* = *37.04)*	NR	Treatment satisfaction was high, with no statistically significant differences between age groups	NR
[Northeastern USA] Walker et al. [86]	210	Naturalistic Study	AN-BP: n = 77 (36.7%) AN-R: n = 54 (25.7%) BN: n = 42 (20.0%) BED: n = 22 (5.2%) OSFED: n = 24 (11.4%) ARFID: n = 2 (1.0%)	CBT-E + DBT 4-h per day, 3 days per week from 2013 to 2015 and up to 4 days per week from 2015 to 2017 *Average of 15.82 (SD* = *13.38) weeks*	EAT-26: Patients who completed four or more IOP weeks demonstrated statistically significant decreases from Week 1 to discharge	NR	NR

ACT: acceptance and commitment therapy; AN: anorexia nervosa; AN-BP: anorexia nervosa, binge-purge type; AN-R: anorexia nervosa, restricting type; ARFID: avoidant/restrictive food intake disorder; BED: binge eating disorder; BMI: body mass index; BN: bulimia nervosa; CBT: cognitive behavioral therapy; CBT-AR: cognitive behavioral therapy for avoidant/restrictive food intake disorder; CBT-E: enhanced cognitive behavioral therapy; C&A: children and adolescents; CGAS: Children’s Global Assessment Scale; DBT: dialectical behavior therapy; EAT-26: Eating Attitudes Test-26; EBW: expected body weight; ED: eating disorders; EDE: Eating Disorder Examination; EDE-Q: Eating Disorder Examination Questionnaire; EDI-2: Eating Disorder Inventory-2; EDNOS: eating disorder not otherwise specified; EFFT: emotion-focused family therapy; EPSI: Eating Pathology Symptoms Inventory; FBT: family-based treatment; FT-ED: family therapy for eating disorders; HBT: home based treatment; HT: home treatment; IBW: ideal body weight; IOP: intensive outpatient program; IPT: inpatient treatment; MANTRA: Maudsley anorexia nervosa treatment for adults; M (SD): mean (standard deviation); NR: not reported; OP: outpatient program; OSFED: other specified feeding or eating disorders; PD: purging disorder; PHP: partial hospitalization program; TAU: treatment as usual: UK: United Kingdom; USA: United States of America; VIOP: virtual intensive outpatient program; W4H: Weight for Height ratio

*This paper used the same participants/dataset as Pauli et al. [54]

**Table 5:** Summary of qualitative findings: positive aspects and challenges/areas for improvement

Treatment programs and author(s)	N	Distribution of participants by diagnosis	Brief treatment description	Brief summary of findings
*Programs for children and adolescents*				
Home-based treatments				
North Buckinghamshire Child and Adolescent Mental Health Service [Aylesbury, UK] Bezance and Holliday [43]	9	Mothers of daughters with AN or EDNOS-AN: n = 9 (100%)	FBT *Varied from 2 to 12 weeks (approximately 8 weeks)*	*Positive aspects:* (i) Source of hope for participants; (ii) reduced carer burden and increased confidence in supporting daughters; (iii) practical and emotional support to restore their own resources and take back control *Challenges/areas for improvement:* (i) Confusion and lack of information around HT and mother's role in treatment; (ii) negative impact of inconsistency between staff members/change in person visiting; (iii) concerns around the impact of HT on other family members, specifically other children; (iv) feelings of insufficient treatment length and fear of relapse post-HT *5 out of 9 adolescents received inpatient treatment following HT, several mothers of these adolescents felt HT had been offered too late
Child and Adolescent Home Treatment Team [Gloucestershire, UK] Clark-Stone et al. [44]	6	AN n = 6 (100%)	FBT Starting with 10 contacts per week and gradually reducing *A period of 6 weeks*	*Positive aspects:* (i) New skills to manage distress at mealtimes; (ii) provided hope and empowerment in families, even following end of intervention *Challenges/areas for improvement:* (i) Personality differences between staff and families created mixed views amongst parents; (ii) most described initial meals with HT team as "awkward", "distressing" and "scary"
Hospital Infantil Universitario Niño Jesús [Madrid, Spain] Morón-Nozaleda et al. [53]	43	Carers of adolescents with AN (restrictive type): n = 30 (50.8%) AN atypical: n = 7 (11.8%) AN (purging type): n = 3 (5.1%) BN: n = 5 (8.5%) ARFID: n = 3 (5.1%) OSFED: n = 11 (18.6%)	CBT + FBT 5 × home visits per week + 2 programmed calls per day *Mean stay of 39.14 days (SD* = *14.47)*	*Positive aspects:* (i) a better understanding of the disease; (ii) newly acquired ability to manage the disease in a natural environment and gain confidence; (iii) participation of all family members; (iv) lack of patient separation from the family environment; (v) greater adaptation to everyday life; (vi) better parental understanding of the sacrifices made by patients; (vii) comfort and reassurance derived from playing an active role in recovery; and (viii) reduced fear and anguish in the face of illness *Challenges/areas for improvement:* Families mostly highlighted difficulties with family logistics
Community-based treatments				
Eating Disorder Intensive Pathway—East London Foundation Trust [London, UK] Komarova [59]	5	NA (clinicians treating children and young people)	FT-ED (Maudsley Approach) + distress tolerance + CBT 3–5 times a week *A period of 6–8 weeks*	*Positive aspects:* (i) flexibility of care (adjusting duration of pathway to needs of patient); (ii) preventing inpatient admissions; (iii) multidisciplinary team interventions; and (iv) the intensity and duration of treatment *Challenges/areas for improvement:* (i) Admission prevention and in-reach; (ii) clinical team; (iii) staffing levels; (iv) staff training; and (v) practical challenges (e.g., room booking)
*Programs for adults*				
Community-based treatments				
Anorexia Nervosa Intensive Treatment Team (ANITT) [Lothian region, UK] Hannon et al. [68]	5	AN-R: n = 4 (80%) AN-BP: n = 1 (20%)	Schema Therapy 2–10 contacts a week *A period of 18-month with 6-month period of reduced-intensity treatment (progress reviews)*	*Positive aspects:* (i) Sense of safety and control and ability to practice change in the 'real world'; (ii) therapeutic alliance providing continuity of reliable, empathic and compassionate relationships and building trust; (iii) collaborative, personalized and flexible treatment planning; (iv) slow pace of change and focus on wellbeing beyond just eating and weight *Challenges/areas for improvement:* Experiencing difficulty when team members with whom participants had built up positive relationships left the team
Yorkshire Centre for Eating Disorders [Leeds, UK] Saeidi et al. [78]	Patients: 6 Clinical team members: 8	AN: n = 6 (100%)	Treatment approach NR 2–7.5 h. per week, up to five visits per week *Length NR*	*Positive aspects:* Patients: (i) Improved quality of life; (ii) accessibility and approachability of staff; (iii) a reduction in the number of hospital admissions, (iv) improved confidence, particularly as they were often involved in planning and reviewing their own care Clinical team: (i) improve access for people with SEEDs to general wards by facilitating admission at an earlier stage; (ii) have a clear plan of action in place for service users before admission; (iii) support staff to manage SEED service users while on the ward
Ladder to the Moon Program [Atlanta-Georgia, USA] Crenshaw [65]	10	AN-BP: n = 2 (20%) BN (binge-purge type): n = 2 (20%) BN (binge type): n = 6 (60%)	Feminist Consciousness 3 × 3-h group therapy sessions per week *A period of 3 weeks to 6 months*	*Positive aspects:* (i) "Acceptance": (i) acceptance of themselves and their bodies; (ii) acceptance of others; and (iii) acceptance of their spirituality (ii) "Making Different Choices": (i) making choices about depending on others; (ii) making choices about how to express feelings of anger or loss of control; and (iii) making choices about experiencing sexuality *Challenges/areas for improvement:* The process of developing a feminist consciousness at times created anxiety producing shifts in thinking
Within Health* [USA] Wolfe et al. [87]	38	NR	Integrative treatment model: ACT, CBT, DBT, interpersonal with experiential modalities (e.g., art therapy, movement) (indicated based on patient presentation) at least 3 h per day; 3–5 days per week *Length NR*	*Positive aspects:* (i) sense of being "seen" and "heard" by providers, fostering authentic connections; (ii) genuine care, empathy, and commitment of providers, promoting trust and support; (iii) creation of a “sense of community” in remote care, facilitating engagement and support *Challenges/areas for improvement:* (i) lack of physical presence and contact, making it harder to connect and build relationships; (ii) technological issues (e.g., bugs, small screen) that hinder smooth communication and engagement; (iii) privacy concerns and distractions in remote settings, affecting the comfort and focus of treatment
*Programs for all age*				
Community-based treatments				
Equilibrio [Colombia] Rodríguez Guarin et al. [85]	Patients: 14 Family members:10 Therapists: 8	AN-R: n = 3 AN-BP: n = 3 AN (atypical) n = 3 BN: n = 6	CBT + Psychodynamic psychotherapy + DBT + Expressive therapy + Art therapy ~ 19 h per week, including individual, group and family sessions *Length is not clear, at least 8 weeks*	*Positive aspects:* (i) being able to stay home and continuity of the treatment, (ii) allowing therapists to observe family dynamics and intervene directly in the home environment, (iii) eliminating the need to travel and for the flexibility it provided around schedules *Challenges/areas for improvement:* (i) privacy, (ii) connection difficulties, (iii) lower level of participation in group sessions, (iv) no physical examination, (v) the need for "more teaching-orientated" methods during the group sessions, (vi) lack of adequate assistance during critical moments before or after meals, affecting adherence to the nutritional regimen * Adherence to sessions was 100% for family members and 90% for patients

ACT: acceptance and commitment therapy; AN: anorexia nervosa; AN-BP: anorexia nervosa, binge-purge type; AN-R: anorexia nervosa, restricting type; ARFID: avoidant/restrictive food intake disorder; BN: bulimia nervosa; CBT: cognitive behavioral therapy; DBT: dialectical behavior therapy; EDNOS: eating disorder not otherwise specified; FBT: family-based treatment; FT-ED: family therapy for eating disorders; NA: not applicable; NR: not reported; SD: standard deviation; OSFED: other specified feeding or eating disorders; UK: United Kingdom; USA: United States of America

^*^This program offers treatment for all age groups, however qualitative outcomes were only focusing on adult patients

As shown in Table 2, the included papers represented a range of study designs with the majority of the papers rated as moderate to low for the quality of evidence for effectiveness. Only one study [70] fell into the highest level of classification (level 1—“experimental designs”) with a pseudo-RCT design. Six studies came under the second level (“quasi-experimental designs”), while the majority were at level 3 (“observational-analytic designs”—17 studies) or at level 4 (“observational-descriptive studies”—9 studies). Similarly, all ten studies reporting evidence on meaningfulness fell into level 3 (“single qualitative study”), and the six studies including evidence for economic evaluation came under the second lowest level of evidence (level 6: “Single economic evaluation of moderate or poor quality”).

#### Quantitative outcomes

##### Programs for children and adolescents

Six sources focusing on HBT [44, 45, 49, 50, 52, 54] and five sources focusing on ICT programs [56–58, 60, 63] reported BMI and/or weight-related outcomes. Furthermore, four studies on HBT [45, 49, 50, 54] and five studies on ICT programs [56–58, 60, 63] assessed outcomes related to ED psychopathology (e.g., binge-purge behaviors, restriction). All of these reported significant improvements in BMI and/or weight related as well as ED psychopathology outcomes. Furthermore, seven of these studies reported follow-up data, with follow-up durations ranging from 1 month to 1 year, presenting continued or maintained improvement over time [49, 50, 52, 56–58, 63].

The number of studies which included outcomes on feasibility and acceptability (e.g., treatment retention, drop out, treatment satisfaction) were fewer, with five studies on HBT programs [45, 49, 50, 53, 54] and two on ICT programs [55, 57] reporting this. These papers reported high levels of satisfaction and good levels of treatment adherence.

Cost-effectiveness outcomes for the treatment programs were presented in four studies on HBT [49, 50, 52, 53] and one study on ICT [63]. These sources reported reduced treatment cost and/or number of/length of hospital stays.

##### Programs for adults

Eight sources focusing on ICT programs [66, 71–76, 80] reported BMI and/or weight related outcomes. Furthermore, one HBT study [64] and eleven ICT studies [66, 69–73, 75, 76, 78, 80, 82] assessed outcomes related to ED psychopathology. Significant improvements were reported for BMI and/or weight as well as ED psychopathology outcomes following treatment, except one case study in a non-underweight patient documented stable BMI over the course of the treatment [73]. Five of these sources also reported follow-up data, with follow-up durations ranging from 1 month to 1 year, describing that improvements either continued or were maintained over time [69–71, 73, 75].

One HBT study [64] and nine ICT studies [69–71, 74, 76, 77, 79, 80, 82] presented feasibility and acceptability outcomes. These studies reported high levels of satisfaction and good levels of treatment adherence, except for one investigating at-home tube feeding for adults with BN, which most participants rated as a difficult experience [64].

Only two ICT studies [77, 78] reported cost-effectiveness outcomes. These studies reported reductions in the treatment cost and/or number of/length of hospital stays.

##### Programs for all age populations

Outcome data for these programs were limited. Among seven sources, there was only one ICT program presenting outcomes on ED psychopathology [86] which reported improvements following treatment. One other study presented feasibility and acceptability outcomes, demonstrating high levels of program adherence. No papers reported cost-effectiveness outcomes for this type of programs.

#### Qualitative outcomes

Qualitative outcomes were reported in nine papers, four on children and adolescents [43, 44, 53, 59] four on adults [65, 68, 78, 87], and one on an all age population [85]. These papers commonly presented both positive aspects and challenges/areas for improvement. Reported positive aspects included increased hopefulness, enhanced understanding of the illness, active involvement in treatment planning, acquisition of new skills, reduced isolation, and decreased hospital admissions. Described perceived challenges/areas for improvement in these papers were logistical and practical difficulties (e.g., room bookings, family logistics, technical/connection difficulties), concerns around the impact of program on other family members and staff turnover and changes during the treatment.

## Discussion

This scoping review investigated ICT and HBT for EDs with a particular focus on conceptualization of these programs, their implementation, and the breadth and nature of the available evidence. The review also identified knowledge gaps and offers guidance for further research on this topic.

Two thirds of the included studies focused on ICT and one third on HBT. Nearly half of all sources targeted children and adolescents, with the remainder focusing on adults or all ages. HBT predominantly targeted children and adolescents with anorexia nervosa (AN), while ICT for this age group targeted individuals with any ED. Only two studies on HBT addressed adults or all age groups. In contrast, ICT was more commonly used among adults and across all ages, in patients with various EDs.

Even though certain aspects of the programs included in this review (e.g., admission and discharge criteria) were not always clearly presented, the majority of the programs aligned with a stepped-care model. These programs were primarily offered as step-up (e.g., moving from outpatient to ICT) or step-down (e.g., transitioning from inpatient to HBT) options, offering flexible levels of care based on patients’ progress and clinical needs. and easing transition from hospital settings to patients' natural environments. These programs also appeared to have the potential to reduce costs, by preventing inpatient admissions or reducing length of hospital stays. Even though ED clinical guidelines [11, 12, 14, 15] recommend using stepped-care treatment models and emphasize maintaining patients’ links with their home and community environments (e.g., social network, education, employment), there are as yet no specific recommendations regarding the use of ICT and HBT for EDs.

Unsurprisingly, this review highlighted differences in psychotherapeutic approaches across age groups. For children and adolescents, ED-focused family therapy (FT-ED) was the most common approach, either on its own or combined with CBT and/or DBT. In contrast, adult and all age programs typically offered combinations of individual psychotherapeutic approaches, such as CBT, DBT, schema therapy or ACT, and optional family involvement. Even though “third-wave” behavioral therapies like DBT and schema therapy are not recommended in clinical guidelines as first-line treatments for EDs, it appears that these approaches are offered in HBT or ICT programs due to promising preliminary evidence of their efficacy [9, 14, 88, 89] and perhaps also, as patients may have tried usual first line treatments previously without success.

Differences in the conceptualization and implementation of these programs can be attributed to several factors. Geographical barriers (e.g., postcode lottery, travel time) also impact the delivery and accessibility of both ICT and HBT outside large urban centres [90–92]. Additionally, limited availability of HBT for adults may stem from the fact that it might be potentially less effective or relevant for this group compared to children and adolescents. Adults may have fewer opportunities to involve family members in the treatment process at home, especially if they live alone or with individuals who are not closely connected. For children and adolescents, hospital admissions can be particularly traumatic, leading clinical teams to prioritize HBT. While family involvement can enhance treatment outcomes, implementing FT-ED and engaging families as an integral part of the process is challenging across all age groups. Geographical barriers, low income, single-parent households, and obligations such as childcare or employment can create significant burdens, making it harder for families to attend treatment sessions and manage tasks like meal preparation and supervision [93].

The available evidence on outcomes of HBTs and ICTs for EDs across different age groups were also mapped. Among 46 sources included, 31 provided quantitative outcomes while nine provided qualitative outcomes. Studies commonly reported improvements in BMI, weight restoration, and eating disorder symptoms (e.g., restriction, binge eating, purging), with some including follow-up data suggesting that these outcomes may be maintained over time. Few studies reported cost-effectiveness outcomes, citing reductions in inpatient admissions and overall healthcare costs. Beyond clinical outcomes, the HBTs and ICTs included in this review were frequently described as feasible and acceptable, with reports of patient and family satisfaction, high program adherence, and low dropout rates. Additional perceived benefits included personalization of care, increased flexibility, greater family involvement, and continuity of care within a familiar environment. Although not directly reported in the included sources, these approaches also have potential to reduce stigma and distress associated with institutionalization, while allowing clinicians to observe family dynamics more naturally, providing insights into home life and relationships [94, 95].

Several studies highlighted different challenges regarding HBTs and ICTs, including inconsistent staff availability, concerns around the impact of HBT on other family members, privacy concerns, technological barriers and lack of physical contact for virtual treatments. In addition to these, geographical aspects and workforce requirements can bring challenges in implementing and delivering these treatments. For instance, staff-to-patient ratios, commutes and transportation costs for both staff and patients are barriers to accessibility, particularly in rural areas with limited provider availability [90]. Another disadvantage could be reduced peer support opportunities in HBTs, which are often a core component of inpatient and day-treatment programs.

Our findings are consistent with observations from other psychiatric populations [25, 29, 30] suggesting that ICTs and HBTs may be potential alternatives to institution-based intensive treatments. However, as methodology of this paper focused on mapping the existing evidence without conducting a synthesis or critical appraisal of efficacy, reported benefits and improvements should be interpreted cautiously. Further evaluation through systematic review and/or meta-analysis would be required to determine the efficacy, acceptability, and cost-effectiveness of these treatments.

### Gaps and recommendations for future work

This review highlights various gaps in the literature on intensive community and home-based treatments for EDs and offers suggestions for future research.

A major gap in the reviewed literature pertains to the description of the treatment programs. Essential details, such as admission and discharge criteria, treatment components and procedures, program duration, weekly number and duration of sessions, were often not clearly specified. Similarly, where interventions which were originally developed for outpatient settings (e.g., FT-ED, CBT) were integrated into programs, information was lacking as to whether these had been adapted for ICT or HBT settings, and if so in what way. Future research would benefit from providing clear definitions of components, and procedures for ICT or HBT programs which are critical for understanding how and why a program was effective (or not) and for whom, improving transparency and comparability, allowing replication, informing policy, guiding practice, and strengthening the evidence base [96, 97].

Additionally, there was a lack of comprehensive reporting on race and ethnicity among the sources included here. Furthermore, only half of the studies included both female and male patients. Notably, reporting of gender identity was almost non-existent outside of the male–female binary. Capturing such information is important, given the growing evidence suggesting that EDs may be more prevalent in gender non-conforming populations [98, 99]. Underreporting of protected or under-served characteristics may be due to regulations in some countries that restrict or discourage the collection of such data. Where possible, future research should improve reporting and increase representation to ensure rigorous evaluation of ICTs and HBTs across diverse groups.

Another notable limitation concerning the available evidence is the scarcity of higher-level evidence. We identified only one RCT protocol [42] and no completed RCTs. This dearth of experimental studies reflects both the recency of interest in HBT and ICT programs and the unique challenges in conducting RCTs within intensive ED treatment settings. These challenges include patient-related (e.g., treatment preferences), service-related, and wider systemic factors [18, 100, 101]. Moreover, the existing literature is also characterized by variability in outcome measures and data collection periods [102]. Large well-designed RCTs of HBT and ICT programs in different ED populations, assessing clinical effectiveness for patients, impacts on families and cost-effectiveness are an obvious next step. In addition, information from large-scale prospectively gathered naturalistic studies of HBT and ICT programmes would also be of value. Such studies, especially if they included genomic and deeply phenotyped clinical information, together with comorbidity and treatment outcome data could lead to advances in personalization of HBT and ICT through allowing appropriate patient stratification and better prediction of clinical outcomes [103].

### Strengths and limitations

To the best of our knowledge this is the first scoping review study systematically investigating non-institutional (i.e., community and home-based) intensive treatments for EDs. Our review provides a comprehensive overview and insight into the extent of literature, characteristics of the programs and their outcomes. Other notable strengths were that the review followed reporting guidelines and used a broad range of evidence sources and methodologies. Moreover, we successfully managed to contact nine lead authors of fourteen studies to clarify some of the missing program details.

The current review also has several limitations. Firstly, although no restrictions were imposed for the publication type or geographical location, the studies included in this review were limited to publications in English. Another limitation was potential exclusion of some sources due to a lack of clarity in reporting of treatment settings and intensity. Finally, some of the outcomes presented in this review were either from the same treatment programs or involved the same patient cohorts in their sample. This overlap may introduce a bias and limit the generalizability and robustness of the conclusions drawn from the review.

## Conclusion

This scoping review showed that available HBT programs are predominantly targeting children and adolescents with anorexia nervosa and used family-focused approaches. ICT programs exhibited greater variability in terms of age, diagnostic populations, and treatment approaches. The evidence base for efficacy, acceptability and cost-effectiveness thus far is limited by a lack of RCTs. However, the available literature, whilst heterogeneous in design, suggests that ICTs and HBTs for EDs may be promising alternatives to traditional institution-based intensive treatments, particularly for improving treatment experiences and reducing treatment costs and hospital admissions. To better understand which programs are the most effective and which approach works best for specific patient populations, future research should focus on conducting higher-quality studies. Such studies would need to includeimproved and consistent reporting of program characteristics and ideally use the same outcome measures across studies. This would also facilitate conduct of comprehensive systematic reviews and meta-analyses.

## Supplementary Information

Additional file1 (DOCX 40 KB)

## Data Availability

Data sharing is not applicable to this article as no new data were created or analyzed in this study.

## Abbreviations

ACT: Acceptance and commitment therapy
AN: Anorexia nervosa
AN-BP: Anorexia nervosa, binge-purge type
ANITT: Anorexia Nervosa Intensive Treatment Team
AN-R: Anorexia nervosa, restricting type
ARFID: Avoidant/restrictive food intake disorder
BED: Binge eating disorder
BMI: Body mass index
BN: Bulimia nervosa
C&A: Children and adolescents
CAMHS: Child and Adolescent Mental Health Services
CBT: Cognitive behavior therapy
CBT-AR: Cognitive-behavior therapy for avoidant/restrictive food intake
CBT-E: Enhanced cognitive behavior therapy
CGAS: Children’s Global Assessment Scale
CRT: Cognitive remediation therapy
DBT: Dialectical behavior therapy
DEBQ: Dutch eating behavior questionnaire
DPT: Day patient treatment
DSM: Diagnostic and statistical manual of mental disorders
EAT-26: Eating attitudes test-26
EBW: Expected body weight
ED: Eating disorder
EDE: Eating disorder examination
EDE-Q: Eating disorder examination questionnaire
EDI-2: Eating disorder inventory-2
EDIP: Eating disorder intensive pathway
EDNOS: Eating disorder not otherwise specified
EFFT: Emotion-focused family therapy
EPSI: Eating pathology symptoms inventory
FBT: Family-based treatment
FT-ED: Family therapy for eating disorders
HBT: Home-based treatment
IBW: Ideal body weight
ICT: Intensive community treatment
IOP: Intensive outpatient program
IPT: Inpatient treatment
JBI: Joanna Briggs Institute
M (SD): Mean (standard deviation)
MANTRA: Maudsley anorexia nervosa treatment for adults
MBSR: Mindfulness-based stress reduction
NA: Not applicable
NR: Not reported
OP: Outpatient program
OSFED: Other specified feeding or eating disorders
PHP: Partial hospitalization program
PRISMA-ScR: Preferred reporting items for systematic review and meta-analysis protocol extension for scoping reviews
PTSD: Post-traumatic stress disorder
RCT: Randomized controlled trial
TAU: Treatment as usual
TF: Tube feeding
UK: United Kingdom
USA: United States of America
VIOP: Virtual intensive outpatient program
W4H: Weight for height ratio
