# Supplementary material for: Intensive community and home-based treatments for eating disorders: a scoping review
Source: J Eat Disord. 2025 Nov 10;13:256. doi: 10.1186/s40337-025-01429-1 (PMC12604378; doi:10.1186/s40337-025-01429-1)
Supplement: Supplementary file 1 — Additional file1 (DOCX 40 KB) [file 40337_2025_1429_MOESM1_ESM.docx]

**Supplemental material 1**

This document provides details of the search terms and strategy used for the main database search.

**PubMed**

| #7 | #5 AND #6 |
| --- | --- |
| #6 | #3 OR #4 |
| #5 | #1 OR #2 |
| #4 | "eating disorder*"[Title/Abstract] OR "anorex*"[Title/Abstract] OR "bulimi*"[Title/Abstract] OR "binge eating*"[Title/Abstract] OR "Avoidant Restrictive Food Intake Disorder" OR"ARFID"[Title/Abstract] |
| #3 | "Anorexia Nervosa"[MeSH Terms] OR "Anorexia"[MeSH Terms] OR "Avoidant Restrictive Food Intake Disorder"[MeSH Terms] OR "Binge-Eating Disorder"[MeSH Terms] OR "Bulimia Nervosa"[MeSH Terms] OR "Bulimia"[MeSH Terms] OR"Feeding and Eating Disorders"[MeSH Terms] |
| #2 | "community care*"[Title/Abstract] OR "community treat*"[Title/Abstract] OR "intensive community*"[Title/Abstract] OR "intensive outpatient*"[Title/Abstract] OR "home care*"[Title/Abstract] OR "home treat*"[Title/Abstract] OR "home feed*"[Title/Abstract] |
| #1 | "Home Care Services"[Mesh] OR "Home Care Services, Hospital-Based"[Mesh] OR "Community Mental Health Services"[Mesh] OR "Community Mental Health Centers"[Mesh] |

**APA PsycInfo**

| #13 | 11 and 12 |
| --- | --- |
| #12 | 7 or 8 or 9 or 10 |
| #11 | 1 or 2 or 3 or 4 or 5 or 6 |
| #10 | (eating adj3 disorder*).mp. |
| #9 | (anorex* or bulimi* or binge eat* or Avoidant Restrictive Food Intake Disorder or ARFID).mp. |
| #8 | (Feeding and Eating Disorder*).mp. |
| #7 | exp Eating Disorders/ |
| #6 | (home adj3 feed*).mp. |
| #5 | (home adj3 treat*).mp. |
| #4 | (home adj3 care*).mp. |
| #3 | (community care* or community treat* or intensive community* or intensive outpatient*).mp. |
| #2 | exp Home Care/ |
| #1 | exp Community Mental Health Services/ or exp Community Mental Health Centers/ |

**Ovid MEDLINE**

| #12 | 10 and 11 |
| --- | --- |
| #11 | 7 or 8 or 9 |
| #10 | 1 or 2 or 3 or 4 or 5 or 6 |
| #9 | (eating adj3 disorder*).mp. [mp=title, book title, abstract, original title, name of substance word, subject heading word, floating sub-heading word, keyword heading word, organism supplementary concept word, protocol supplementary concept word, rare disease supplementary concept word, unique identifier, synonyms, population supplementary concept word, anatomy supplementary concept word] |
| #8 | (anorex* or bulimi* or binge eating* or Avoidant Restrictive Food Intake Disorder or ARFID).mp. [mp=title, book title, abstract, original title, name of substance word, subject heading word, floating sub-heading word, keyword heading word, organism supplementary concept word, protocol supplementary concept word, rare disease supplementary concept word, unique identifier, synonyms, population supplementary concept word, anatomy supplementary concept word] |
| #7 | exp "Feeding and Eating Disorders"/ |
| #6 | (home adj3 feed*).mp. [mp=title, book title, abstract, original title, name of substance word, subject heading word, floating sub-heading word, keyword heading word, organism supplementary concept word, protocol supplementary concept word, rare disease supplementary concept word, unique identifier, synonyms, population supplementary concept word, anatomy supplementary concept word] |
| #5 | (home adj3 treat*).mp. [mp=title, book title, abstract, original title, name of substance word, subject heading word, floating sub-heading word, keyword heading word, organism supplementary concept word, protocol supplementary concept word, rare disease supplementary concept word, unique identifier, synonyms, population supplementary concept word, anatomy supplementary concept word] |
| #4 | (home adj3 care*).mp. [mp=title, book title, abstract, original title, name of substance word, subject heading word, floating sub-heading word, keyword heading word, organism supplementary concept word, protocol supplementary concept word, rare disease supplementary concept word, unique identifier, synonyms, population supplementary concept word, anatomy supplementary concept word] |
| #3 | (community care* or community treat* or intensive community* or intensive outpatient*).mp. [mp=title, book title, abstract, original title, name of substance word, subject heading word, floating sub-heading word, keyword heading word, organism supplementary concept word, protocol supplementary concept word, rare disease supplementary concept word, unique identifier, synonyms, population supplementary concept word, anatomy supplementary concept word] |
| #2 | exp Home Care Services/ |
| #1 | exp Community Mental Health Centers/ or exp Community Mental Health Services/ |

**Web of Science**

| Query number | Search term |
| --- | --- |
| #4 | #1 AND #2 |
| #3 | #1 AND #2 |
| #2 | TS=((community NEAR/3 "care*”) OR (community NEAR/3 "treat*”) OR (intensive NEAR/3 "community*”) OR (intensive NEAR/3 "outpatient*”) OR (home NEAR/3 "care*”) OR (home NEAR/3 "treat*”) OR (home NEAR/3 " feed*”)) |
| #1 | TS=("eating disorder*" OR "anorex*" OR "bulimi*" OR "binge eat*" OR "Avoidant Restrictive Food Intake Disorder" OR "ARFID") |

**Supplemental Material 2**

This document presents the data charting form developed for this scoping review.

| **Data Item/Category** | **Extracted Information** |
| --- | --- |
| Author(s) |  |
| Publication Year |  |
| Country |  |
| Sample Size (N) |  |
| Mean/Median Age at Baseline |  |
| Sex, Gender, and Gender Identity |  |
| Race and Ethnicity |  |
| Socioeconomic Status |  |
| Treatment Setting |  |
| Distribution of Participants by Diagnosis |  |
| Study Design |  |
| Data Type |  |
| Level of Evidence for Effectiveness |  |
| Level of Evidence for Meaningfulness |  |
| Level of Evidence for Economic Evaluation |  |
| Treated Population |  |
| Admission Criteria |  |
| Underlying Treatment Model(s) |  |
| Meal Support / Supervised Meal |  |
| Length and Intensity |  |
| Delivery Mode |  |
| Professional(s) Delivering the Intervention |  |
| Carer / Family Involvement |  |
| ED-related Outcomes (e.g., BMI, Symptoms) |  |
| Feasibility and Acceptability Outcomes |  |
| Cost-effectiveness Outcomes |  |
| Brief Summary of Qualitative Findings |  |
| Additional Notes |  |

**Supplemental Material 3**

This document presents citations and abstracts of papers that could meet the inclusion criteria but were unable to be assessed due to the unavailability of English full texts.

Citation: Piavaux, L., Pareaz, D., & Mieville, M. (1976). Home-treatment of complex anorexia in infants. *Revue de Neuropsychiatrie Infantile et d'Hygiene Mentale de l'Enfance, 24*(1-2), 47–56. [French]

Abstract: Illustrates with a case history a method of home treatment of young mothers who exhibit a pathological structure of the pregenital type and who have a child suffering from complex anorexia. The special features of an intensive relationship fostered in the home render it possible to make use of the regressive position through which the mother passes during this relationship by referring to the early frustrations she has experienced. As a result of this approach, the mother begins to treat the child warmly and adequately. An attempt to standardize this therapeutic approach is being made.

Citation: Cuntz, U. (2015). The development of the German health system in regard to treatment of eating disorders. *Psychotherapie, Psychosomatik, medizinische Psychologie*, *65*(1), 5-7. doi: 10.1055/s-0034-1394409. [German]

Abstract: The ongoing change in the German health care system challenges all those dealing scientifically with the treatment of eating disorders. The process of change provides an opportunity to rethink the entire range from outpatient treatment to intensive residential treatment of mental disorders. Improving trans-sectoral medical care for eating disorders could thereby play a leading role. The problem of unsatisfactory long-term courses in view of the high mortality in anorexia nervosa, cannot be met only by successful weight restitution but by an adaptive approach for long term maintainment of a healthy body weight.

Citation: Herpertz-Dahlmann, B., Dahmen, B., Zielinski-Gussen, I. M., & Seitz, J. (2024). Neue Aspekte in der Ätiologie und Therapie der jugendlichen Anorexia nervosa–ein postuliertes biopsychosoziales Modell und die Auswirkungen der COVID-19-Pandemie. *Bundesgesundheitsblatt-Gesundheitsforschung-Gesundheitsschutz*, *67*(4), 400-408. doi: 10.1007/s00103-024-03856-y. [German]

Abstract: Anorexia nervosa is one of the most frequent chronic disorders of adolescence associated with a high mortality. During the COVID-19-pandemic, the number of hospitalized children and adolescents with anorexia nervosa significantly increased. This article outlines new research findings to decode the etiology of this serious disorder, especially a genetic disposition and changes of metabolism. Against the background of increasing rates during the COVID-19 pandemic, the importance of the gene-environment interaction is discussed, and new treatment forms are described. Besides the development of new biological treatment strategies, there is also some important progress in psychotherapeutic interventions. Carers should always be integrated when treating children and adolescents with anorexia nervosa, which is especially emphasized in the new "home treatment" setting. The new concept of anorexia nervosa as a metabo-psychiatric disorder gives us hope for new research ideas and treatment strategies in this often-debilitating disorder of childhood and adolescence.

Citation: Altdorf, S., Dempfle, A., Heider, K., Seitz, J., Herpertz-Dahlmann, B., & Dahmen, B. (2022). Eltern als Ko-Therapeuten im home treatment bei adoleszenter Anorexia nervosa –Wirkfaktoren und Mechanismen [Parents as Co-Therapists in Home Treatment for Adolescents with Anorexia Nervosa - Factors and Mechanisms]. *Praxis der Kinderpsychologie und Kinderpsychiatrie*, *71*(5), 467–486. <https://doi.org/10.13109/prkk.2022.71.5.467> [German]

Abstract: Caring for a child with anorexia nervosa (AN) puts a strain on many parents. At the same time, actively involving the parents in treatment to increase their skills to manage the disorder is important, as it seems to improve the child's prognosis. Home treatment requires the parents to be particularly involved. The aim of this study was to assess parental burden and caregiving skills and the association of these factors with the child's AN pathology in the course of a multidisciplinary home treatment (HoT). After 4 to 8 weeks of inpatient treatment, 22 adolescent patients with AN received home treatment with intensive involvement of their parents as co-therapists. Caregiving burden and caregiving behavior and the symptom severity of the child's AN were assessed using standardized questionnaires on admission to the hospital, at discharge from the HoT, and 1 year after admission (Eating Disorder Inventory-2 (EDI-II), Beck Depression Inventory-2 (BDI-II), Accommodation and Enabling Scale for Eating Disorders (AESED), Eating Disorders Symptom Impact Scale (EDSIS)). Parental burden was reduced and the parents' ability to manage their child's AN improved after the step-down treatment from inpatient treatment to home treatment and was also associated with lower eating disorder-specific psychopathology of the patients. HoT as a treatment setting does not seem to burden the parents as co-therapists and is associated with an improvement in skills in dealing with the child's AN.

Citation: Wilken, M., Mink, C. M., & Böhme, J. (2023). Zur Notwendigkeit evidenzbasierter Behandlung frühkindliche Sondendependenz – Ein Review zur Analyse von Störungsbild und Behandlung basierend auf dem PICO-Schema [The Need of Evidence-Based Treatment of Early-Onset Feeding Tube Dependency: A Pico- Framework Based Analytic Review of Clinical Features and Treatment of Feeding Tube Dependency]. *Praxis der Kinderpsychologie und Kinderpsychiatrie*, *72*(6), 529–551. <https://doi.org/10.13109/prkk.2023.72.6.529> [German]

Abstract: Feeding Tube Dependency is a constant increasing perinatal mental health condition, with estimated 350 new cases per year in Germany. The early onset feeding tube dependency is the consequence of a generalized food aversion. To establish an evidence-based nationwide treatment plan, relevant research from the past twenty years were narratively reviewed. Feeding tube dependency is an international increasing mental health condition, with a high symptom persistence and a low spontaneous remission. The generalized food aversion would prevent the transition to oral feeding. The treatment protocols, supported by the German Health System, based on low-frequency individual treatment and intensive inpatient treatments are not supported by the most recent evidence. In treatment outcome research more promising and effective programs can be distinguished from ineffective programs. As a result, treatment which are designed as intensive treatment, home-based or inpatient and are psychodynamic based are most effective. Day-clinic and behavioral modification programs are not or low in treatment effectiveness. The German Health System approach to assist families with feeding tube dependency is not evidence-based. A new structure of treatment is imperatively required.

Citation: Boswell, J. F., Thompson-Brenner, H., Oswald, J. M., Brooks, G. E., & Lowe, M. (2018). The intersection of implementing evidence-based psychotherapy and practice-oriented research/la intersección de la implementación de la psicoterapia basada en la evidencia y la investigación orientada por la práctica. *Revista Argentina de Clínica Psicológica*, *27*(II), 136. <https://doi.org/10.24205/03276716.2018.1063> [Spanish]

Abstract: Residential and other intensive treatment programs for eating disorders (EDs) do not commonly utilize evidence-based interventions coordinated across multi-modal treatment elements. In addition, there is a pressing need to learn more about effective (and ineffective) evidence-based intervention implementation processes and outcomes in service delivery organizations. This ongoing diverse mental health care stakeholder practice-oriented research (POR) “case study” describes the implementation of evidence-based psychotherapy for severe EDs and co-occurring emotional disorders across a network of residential, day hospital, and intensive outpatient treatment programs. Based on stakeholder input, data collection has been embedded throughout the implementation process, spanning clinician-trainee attitudes, group facilitator adherence and competence, and patient outcomes. This paper traces the history of this implementation and POR effort across multiple phases, while integrating theory and research from implementation science, as well as routinely collected data from this effort. Finally, lessons from this ongoing case study may inform future efforts (both internal and external to this specific context) to implement evidence-based strategies in residential/intensive outpatient settings

Citation: Rigaud, D., Pennacchio, H., Roblot, A., Jacquet, M., Tallonneau, I., & Verges, B. (2009). Efficacité de la nutrition entérale à domicile chez 60 malades ayant une anorexie mentale. [Tube feeding at home in anorexia nervosa patients *] La Presse Médicale*, *38*(12), 1739-1745. Doi: 10.1016/j.lpm.2009.04.009. [French]

Abstract: Introduction: In the aim to explore the efficacy of tube feeding (TF) in ambulatory anorexia nervosa (AN) patients, we prospectively treated 60 AN patients by tube feeding (TF) at home, using a nasogastric tube. Methods: Nutritional (clinical, biological) and psychological (Hamilton and Beck scores) markers were assessed before and after 2 months of NEAD. Results: While the patient's body weight was decreasing during the previous 2 months, it significantly increased (P<0.001) during the 2-month TF, both in the restrictive and the binge/purging form: +3,42 +/- 2,39 kg in the restrictive ones and +2,82 +/- 2,17 kg in the binge/purging ones. Patients were rapidly (48 h) and frequently abstinent from binge/purging during TF: 90% had no more binge/purging episodes (P<0.0001). TF did not worse the eating behavior, and did increase neither anxiety nor depressive levels (Hamilton and Beck scores). Biological nutritional markers were normal before TF and remained so (2nd month), except haptoglobin which was low before and reached normal range during TF (P<0,01). Conclusion: Ambulatory TF seems to be useful in AN patients at home. This could permit to avoid hospitalization, but needs to be confirmed by a randomized trial.

Citation: Michler P, Wolter-Flanz A, Linder M. trEATit - Intensive ambulante Gruppentherapie von Jugendlichen mit Essstörungen. [Intensive outpatient group treatment for adolescents with eating disorders]. *Praxis der Kinderpsychologie und Kinderpsychiatrie. 2007* ;56(1):19-39. DOI: 10.13109/prkk.2007.56.1.19. [German]

Abstract: We present an intensive outpatient group treatment for girls with eating disorders (anorexia nervosa, bulimia nervosa, binge eating disorder) additionally to/instead of inpatient treatment or individually treatment by psychotherapists. The therapy concept is primarily behaviour therapy oriented, encouraging the self-management-abilities of the patients thereby learning self-determination and responsibility in dealing with their illness. The slow-open group concept provokes group cohesion, solidarity and support among girls, who share similar age-related development-stages and eating disorders. Other than cognitive behaviour therapy and the principles of self-management we use client-centered therapy, art-, dance- and nutritional therapy. For each patient an individual treatment plan is adapted depending on age, individual symptoms, problems and motivation. Each member of the group has to accept defined group rules during the group sessions. The group takes place twice a week and on one Saturday per month. The adolescents stay in their social environment. Transfer of therapeutic success into daily life therefore is immediate and longlasting. Duration of therapy is between four months and one year, longer only in complex cases. Parallel to the parent/patient cooperation a parental psycho-educative group is available.

Citation: Delaunay, A. L., Gérardin, P., & Godart, N. (2019). Prise en charge en hôpital de jour des adolescents présentant un trouble des conduites alimentaires: revue de la littérature internationale et état des lieux en France. *Neuropsychiatrie de l'Enfance et de l'Adolescence*, *67*(4), 203-212. [French]

Abstract: The French Health Authorities recommendation of 2010 about eating disorder patient care advised for a multi-disciplinary approach adapted to patient’s needs and with different levels of outpatient treatment, inpatient treatment and day-care hospital programs. Objective of the study. — To establish an international literature review and to make a state of knowledge of patient care in day-care hospital program settings for adolescents with eating disorders by a national survey: existing care, modalities and care programs. Method. — The bibliographic search was done according to the Prisma method (23 initial articles and 9 selected articles published between 2003 and 2015). A national survey was sent via email. All 2015 FFAB indexed structures have been contacted. In overall, 62 structures claimed to have a day-care hospital program treating adolescent eating disorder patients. The existing literature promotes the day-care hospital program benefits: global patient evaluation, care grading, intensive outpatient treatment avoiding continued inpatient treatment, post-inpatient treatment handover to step up outpatient treatment, inpatient treatment preparation. Results. — Fifteen of the 62 finally replies stated they offered a day-care hospital program for adolescent eating disorder patients (only five with a specialized patient care for eating disorders), representing 77 beds in day-care hospital settings. All of them treat patients with anorexia nervosa, 73,3% bulimia nervosa and 26,7% hyperphagia. Patient age varies between 6 and 40 years. The patient care weekly frequency varies from 1 to 5 half-days per week. A total of 26,7% follows an intensive care of 5 days per week. Conclusion. — It appears crucial that, in the coming years, France has to set up dedicated daycare hospitals for adolescents, such as those found in Canada, Germany and Great Britain. With a real efficacy, while limiting costs, they allow to smoothly adapt patient care with efficiency for patients while preserving their social insertion.
